# Enhancing Digital Twin Fidelity Through Low-Discrepancy Sequence and Hilbert Curve-Driven Point Cloud Down-Sampling

**DOI:** 10.3390/s25123656

**Published:** 2025-06-11

**Authors:** Yuening Ma, Liang Guo, Min Li

**Affiliations:** School of Mathematics and Statistics, Shandong University, Wen Hua Xi Road 180, Weihai 264209, China; yuening_ma@mail.sdu.edu.cn (Y.M.); minli@sdu.edu.cn (M.L.)

**Keywords:** low-discrepancy sequence, Hilbert curve, point cloud down-sampling

## Abstract

This paper addresses the critical challenge of point cloud down-sampling for digital twin creation, where reducing data volume while preserving geometric fidelity remains an ongoing research problem. We propose a novel down-sampling approach that combines Low-Discrepancy Sequences (LDS) with Hilbert curve ordering to create a method that preserves both global distribution characteristics and local geometric features. Unlike traditional methods that impose uniform density or rely on computationally intensive feature detection, our LDS-Hilbert approach leverages the complementary mathematical properties of Low-Discrepancy Sequences and space-filling curves to achieve balanced sampling that respects the original density distribution while ensuring comprehensive coverage. Through four comprehensive experiments covering parametric surface fitting, mesh reconstruction from basic closed geometries, complex CAD models, and real-world laser scans, we demonstrate that LDS-Hilbert consistently outperforms established methods, including Simple Random Sampling (SRS), Farthest Point Sampling (FPS), and Voxel Grid Filtering (Voxel). Results show parameter recovery improvements often exceeding 50% for parametric models compared to the FPS and Voxel methods, nearly 50% better shape preservation as measured by the Point-to-Mesh Distance (than FPS) and up to 160% as measured by the Viewpoint Feature Histogram Distance (than SRS) on complex real-world scans. The method achieves these improvements without requiring feature-specific calculations, extensive pre-processing, or task-specific training data, making it a practical advance for enhancing digital twin fidelity across diverse application domains.

## 1. Introduction

Light Detection and Ranging (LiDAR) technology is essential for creating digital twins—high-fidelity virtual representations of physical environments. However, the massive point clouds generated by modern LiDAR systems, often containing millions or billions of points, create a fundamental computational bottleneck that impedes the efficient creation and utilization of these digital models [1,2]. Down-sampling techniques, which reduce the density of point clouds while preserving key features, are therefore critical for balancing computational efficiency with model fidelity [3]. The choice of down-sampling method significantly impacts the quality and usability of the resulting digital twin, with inadequate methods potentially compromising essential geometric features, spatial relationships, and surface characteristics that are vital for downstream applications [4,5].

To mitigate these challenges, down-sampling techniques are crucial. By reducing the density of point clouds while preserving key features, down-sampling lowers the computational load and enables faster processing [3]. This, in turn, reduces power consumption, making digital twin generation and maintenance more sustainable [6]. The choice of down-sampling method significantly impacts the fidelity of the resulting point clouds. Common approaches include Simple Random Sampling, which is simple but vulnerable to randomness; Voxel Grid Down-sampling, which can regularize density but struggles to preserve sharp edges; feature-preserving techniques, which are effective but computationally expensive; Farthest Point Sampling, which enforces uniform spacing but tends to lose details in high-density regions; and learning-based methods, which can achieve state-of-the-art results but require large training datasets.

Despite the variety of existing techniques, a fundamental challenge remains: achieving an optimal balance between computational efficiency, preservation of salient geometric features, and faithful representation of the point cloud’s overall spatial distribution [4,5].

This paper addresses this gap by proposing a novel down-sampling approach, LDS-Hilbert, driven by Low-Discrepancy Sequences (LDS) combined with Hilbert curve ordering. While LDS havs been explored for point cloud index selection [7], and space-filling curves (SFCs) have been utilized for points encoding and sorting [8,9], our method introduces a distinct synergistic framework. It uniquely first establishes strong, global 3D spatial coherence by directly mapping the entire point cloud with a Hilbert curve, and then employs an LDS-derived reordering mechanism for a globally uniform yet structure-aware selection from this coherent 1D sequence. Leveraging the inherent uniformity properties of LDS and the superior locality-preserving characteristics of Hilbert curves, our method aims to generate a down-sampled point cloud that better preserves both the global structure and local details of the original data compared to existing methods. By ensuring a more uniformly distributed yet structure-aware sampling, the LDS-Hilbert approach can potentially enhance the fidelity of digital twins constructed from the down-sampled data.

Our extensive experiments demonstrate that the proposed LDS-Hilbert method consistently outperforms traditional down-sampling approaches (SRS, FPS, Voxel) across diverse geometric data and evaluation metrics. Notably, it achieves significant error reductions in parametric surface fitting and superior shape and feature preservation in mesh reconstruction tasks, particularly for complex CAD models and real-world laser scans, without requiring feature-specific calculations or training data.

The key contributions of this work are as follows:

1. **A novel synergistic down-sampling framework:** We propose LDS-Hilbert, a method that uniquely combines Hilbert curve ordering for spatial coherence with Low-Discrepancy Sequences (LDS)-based reordering for globally uniform and representative point selection.
2. **Enhanced fidelity for digital twins:** We demonstrate through comprehensive experiments that LDS-Hilbert significantly improves the fidelity of digital twins constructed from down-sampled point clouds, excelling in both parametric parameter recovery and mesh reconstruction accuracy across various object types.
3. **Robustness and practicality:** The proposed method is deterministic, requires minimal parameter tuning beyond the target sample size, and shows strong performance on noisy real-world scan data, making it a practical solution for diverse applications.
4. **Insight into ordering strategies:** Through ablation studies (LDS-Hilbert vs. LDS-PCA), we provide insights into the importance of locality-preserving spatial ordering (via the Hilbert curve) as a precursor to LDS-based sampling, especially for complex geometries and larger datasets.

The remainder of this paper is organized as follows: Section 2 reviews related work. Section 3 details the proposed LDS-Hilbert methodology. Section 4 presents the experimental setup and results. Finally, Section 5 concludes the paper and discusses future work.

## 2. Literature Review

### 2.1. Point Cloud Down-Sampling Techniques

Point cloud down-sampling is a fundamental pre-processing step in 3D data processing, essential for managing the computational burden associated with large, dense datasets captured by technologies like LiDAR. The primary objective is to reduce the number of points from an initial set while preserving the essential geometric information and structural fidelity required for subsequent tasks, such as digital twin creation, reverse engineering, object recognition, scene reconstruction, or robotic navigation [10,11]. This section surveys established and contemporary down-sampling techniques, categorizing them based on their core strategies, while acknowledging the inherent overlaps and prevalence of hybrid approaches.

#### 2.1.1. Random Sampling Methods

Conceptually the simplest approach, Random Sampling involves selecting a subset of points purely at random from the original point cloud [12]. Its main advantages are implementation ease and high computational efficiency. However, its stochastic nature ignores the spatial distribution and structural importance of points. This can lead to uneven point density, the potential loss of critical geometric features (especially in complex regions), and insufficient coverage, rendering it unsuitable for high-precision applications demanding high fidelity [13].

#### 2.1.2. Spatially Structured Methods

To address the lack of spatial awareness in random methods, spatially structured techniques impose a regular or adaptive partitioning of the 3D space.

- **Voxel Grid Down-sampling**: This widely adopted method discretizes the space encompassing the point cloud into a uniform grid of cubic cells (voxels) [14]. Within each voxel containing points, a single representative point is retained, often the centroid or the point nearest the centroid. This guarantees a minimum spatial separation, effectively regularizes point density, and preserves the coarse structure. However, its primary limitation lies in potential oversimplification, particularly the loss of fine details when multiple significant points fall within a single voxel [4]. The method’s performance is highly sensitive to the chosen voxel size: too large, and details are lost; too small, and the reduction ratio diminishes. Recent work explores dynamic or adaptive voxel sizes to mitigate these issues [4].
- **Octree-based Down-sampling**: Employing a hierarchical data structure, octree methods recursively subdivide the 3D space into eight octants, adapting the partitioning depth based on the local point density [15,16]. Points within leaf nodes are typically represented by a single point (e.g., centroid). This allows for more adaptive sampling than uniform voxel grids, better handling variations in point density. However, implementation is more complex, and performance depends on criteria like the maximum tree depth or points per leaf node, which still require careful tuning to balance detail preservation and computational efficiency [17].

#### 2.1.3. Clustering-Based Methods

Leveraging the spatial distribution of points, clustering-based approaches group nearby points into clusters using algorithms like k-means or DBSCAN [18]. A representative point (e.g., cluster centroid) is then selected from each cluster. This strategy adapts well to non-uniform densities, preserving dense regions while potentially discarding isolated points. However, it is sensitive to clustering parameters (k-value, distance thresholds) and algorithm choice, which can affect performance on varying point cloud structures. Furthermore, the computational cost, especially for iterative algorithms like k-means, can be significant for very large datasets [10].

#### 2.1.4. Feature-Preserving Methods

These techniques prioritize the retention of points deemed geometrically significant, crucial for preserving the shape’s salient characteristics.

- **Curvature/Normal-based Sampling**: Points located in regions of high surface curvature or significant surface normal variation are assigned higher importance and are preferentially retained [19]. Such points often correspond to edges, corners, or areas of intricate detail. While effective at preserving sharp features critical for accurate modeling [20], these methods are computationally more intensive due to the need for local neighborhood analysis to estimate geometric properties. They can also be sensitive to noise, which might be misinterpreted as high curvature, and require careful parameter tuning (e.g., neighborhood size, curvature thresholds). Recent efforts focus on balancing feature preservation with achieving a more uniform point distribution [21].
- **Structure-aware/Salient-point Sampling:** This category encompasses methods that identify important points based on various geometric descriptors or structural roles. Recent approaches may use advanced techniques like Gaussian processes on Riemannian manifolds [22] or implicitly identify salient points within learning frameworks focused on reconstruction quality [23].

#### 2.1.5. Learning-Based Methods

Representing the state-of-the-art frontier, deep learning methods train neural networks to learn optimal sampling strategies. These can be broadly categorized as follows:

- Generative vs. Score-Based: Some networks directly generate the coordinates of the down-sampled points, while others assign an importance score to each original point, followed by a selection process (e.g., top-k scoring points) [24].
- Task-Agnostic vs. Task-Specific: Task-agnostic methods aim for general geometric fidelity, often optimized via reconstruction losses [24] or leveraging distilled 2D information [5]. Task-specific methods optimize sampling for downstream applications like object detection [25], classification [26], registration [11], or surface reconstruction [23].
- Advanced Architectures: Techniques like attention mechanisms and transformers are increasingly employed to capture complex inter-point relationships for more informed sampling [24,25]. Methods also address challenges like imbalanced data distributions [27].

While powerful and adaptable, learning-based methods typically require substantial labeled training data, significant computational resources for training, and their generalization to novel object types or sensor characteristics must be carefully validated [4].

#### 2.1.6. Hybrid Methods

Recognizing that no single strategy is universally optimal, hybrid methods combine elements from different categories to leverage their respective strengths. Examples include using clustering with feature extraction [10] or combining learned sampling strategies with traditional pooling mechanisms [27].

#### 2.1.7. Summary and Motivation

While numerous down-sampling techniques exist, each presents trade-offs between computational efficiency, data reduction ratio, and the preservation of geometric fidelity. Random methods are fast but structurally naive. Voxel and octree methods enforce spatial constraints but can lose details or require careful parameterization. Clustering adapts to density but is parameter-sensitive. Feature-based methods preserve details but are computationally heavier and noise-sensitive. Learning-based methods offer high potential but demand significant data and computational resources, with potential generalization issues.

For digital twin applications demanding high fidelity, these limitations become critical. An ideal down-sampling method for these domains should effectively reduce the data volume while robustly preserving both fine-grained surface details and the overall global structure, maintain the original point cloud’s spatial density variations where meaningful, and remain computationally tractable without extensive training phases. The persistent challenges and trade-offs associated with existing methods motivate the exploration of alternative paradigms.

This paper investigates (1) Low-Discrepancy Sequences, a mathematically grounded approach focused on spatial uniformity, and (2) the Hilbert curve, a mathematically grounded method to map a multi-dimensional space (like 2D or 3D) onto a 1D path while preserving spatial locality, to address this gap, proposing a novel Low-Discrepancy-Sequences-based method designed to enhance digital twin fidelity by generating a more representative and structurally sound down-sampled point cloud.

### 2.2. Low-Discrepancy Sequences and Quasi-Monte Carlo Sampling

Low-Discrepancy Sequences (LDS), also known as quasi-random sequences, offer a deterministic alternative to pseudo-random number sequences (PRNGs), designed to minimize clustering and improve spatial uniformity [28]. The uniformity of a point set is formally quantified by its discrepancy  [29,30]. LDS are mathematically constructed to achieve low discrepancy, ensuring points fill space more evenly than typical PRNGs [31,32,33,34].

The primary application of LDS is in Quasi-Monte Carlo (QMC) methods [28,35]. Beyond numerical integration, their inherent uniformity makes LDS valuable for various sampling tasks. For instance, in selecting a smaller, representative subset from a larger dataset, LDS can guide selection for better spatial coverage than random selection [36]. Xu et al. [7] explored using LDS-generated indices for point cloud down-sampling after an initial PCA-based ordering. While this demonstrates the potential of LDS for structured subset selection, the efficacy of such an approach heavily relies on the initial ordering’s ability to capture the most relevant spatial characteristics; PCA, by focusing on global variance, may not always optimally preserve the local geometric details and intricate manifold structures crucial for high-fidelity reconstruction, especially in complex point clouds. LDS are also widely used in computer graphics, signal processing, and experimental design [36,37,38,39,40]. In essence, for tasks requiring evenly spread representative points, LDS offer a principled approach [41,42].

### 2.3. Hilbert Curve for Spatial Coherence

Beyond primary down-sampling strategies, the method used to structure or order a point cloud prior to sampling significantly influences the outcome. Space-Filling Curves (SFCs) map multi-dimensional data to a 1D sequence while attempting to preserve spatial locality [43,44]. The Hilbert curve is renowned for its superior locality-preserving properties compared to alternatives like the Z-order curve (used in [9]), minimizing large jumps in the 1D sequence for points close in the original *d*-space [45,46,47].

This property has been exploited for efficient spatial indexing [47] and adapting sequential deep learning models for point cloud analysis [48]. For instance, Chen et al. [8] introduced HilbertNet, which applies 2D Hilbert curves slice-wise to Z-axis partitioned voxels for efficient 2D convolutional processing. While innovative for dimensionality reduction, this voxel-based, slice-by-slice 2D Hilbert mapping can lead to loss of fine-grained point cloud details and may not optimally preserve overall 3D spatial locality. Separately, Li et al. [9] proposed PointSCNet, which employs Z-order curve coding on an initially sampled (e.g., via FPS) and grouped point set, followed by an equal-interval sampling along the Z-ordered sequence to select points for feature learning in classification and segmentation tasks. While Z-order provides some geometric ordering, its locality preservation is generally inferior to Hilbert curves. Moreover, the equal-interval sampling can be susceptible to aliasing if its stride resonates with periodic patterns in the data. Furthermore, PointSCNet primarily targets feature learning from sparse point sets. Its efficacy in preserving the comprehensive geometric detail required for high-fidelity digital twins from large-scale, dense point clouds remains unexplored.

The challenge in point cloud down-sampling lies in effectively leveraging these mathematical tools for optimal geometric fidelity. While LDS ensures uniform selection based on indices, its success hinges on the quality of the initial data ordering. Similarly, while Hilbert curves provide excellent spatial ordering of the entire point cloud, a subsequent naive or simplistic sampling from this order may not be optimal for capturing both global distribution and local detail. This paper investigates how a direct 3D Hilbert curve ordering of the raw point cloud, preserving its detailed geometric structure without initial voxelization or aggressive pre-sampling, can serve as a robust foundation. We then explore how this globally coherent, locality-preserving 1D sequence can be synergistically combined with LDS principles to achieve a down-sampled set that is both comprehensively representative and rich in local features.

## 3. Proposed Method

Throughout this paper, we use the following notation conventions: Calligraphic letters (e.g., P) denote point sets; bold lowercase letters (e.g., p) represent individual points or vectors; bold uppercase letters (e.g., M) indicate meshes or matrices; and non-bold lowercase letters (e.g., *d*) represent scalar values. Subscripts indicate indices (e.g., $p_{i}$ is the *i*-th point) or specific subsets (e.g., $P_{\mathrm{sorted}}$ is the sorted point cloud), while superscripts denote alternative versions (e.g., $P^{\prime }$ is the down-sampled point cloud).

We formulate the research problem as minimizing the discrepancy between the reconstructed digital twin and the true underlying surface:

$$\min_{f}D\left(M^{\prime },M_{\mathrm{true}}\right)=\min_{f}D\left(g\left(f\left(P\right)\right),M_{\mathrm{true}}\right)$$

where *f* is the down-sampling function, *g* is the reconstruction function, P is the original point cloud, $M^{\prime }$ is the reconstructed digital twin, and $M_{\mathrm{true}}$ is the true underlying surface.

### 3.1. Algorithm Description

Our algorithm operates in three phases:

1. Ordering with Hilbert Curve Mapping to create spatial coherence.
2. LDS-driven reordering to ensure uniform coverage.
3. Contiguous subsequence selection to preserve local feature relationships.

Algorithm 1 summarizes the process of the proposed point cloud down-sampling method. An illustration of the proposed method can be found in Appendix A.
**Algorithm 1** LDS-Hilbert: A point cloud down-sampling method**Require:** Original point cloud $P={\left\{p_{i}\right\}}_{i=1}^{N}\subset R^{d}$ (d≥2), Target size *M* (M<N)

**Ensure:** Down-sampled point cloud $P^{\prime }={\left\{p_{j}^{\prime }\right\}}_{j=1}^{M}$
  1:**Step 1:** Initial Ordering via Hilbert Curve Mapping  2:N←|P|  3:$P_{\mathrm{norm}}\leftarrow \emptyset$  4:**for** j←1tod **do**  5:     $\min_{j}\leftarrow \min_{x\in P}x_{j}$  6:      ${\mathrm{range}}_{j}\leftarrow \left(\max_{x\in P}x_{j}\right)-\min_{j}$  7:**end for**  8:**for** $p_{i}\in P$ **do**  9:      $p_{\mathrm{norm},i}\leftarrow \mathrm{vector}\;\mathrm{where}\;p_{\mathrm{norm},i,j}=\left(p_{i,j}-\min_{j}\right)/{\mathrm{range}}_{j}\;\mathrm{for}\;j=1\ldots d$10:      $P_{\mathrm{norm}}\leftarrow P_{\mathrm{norm}}\cup \left\{p_{\mathrm{norm},i}\right\}$11:**end for**12:$s\leftarrow \lfloor \log_{2^{d}}\left(N\right)+0.5\rfloor$13:$H_{s}\leftarrow \mathrm{ConstructHilbertCurve}\left(s,d\right)$14:$D_{\mathrm{Hilbert}}\leftarrow \mathrm{array}\;\mathrm{of}\;\mathrm{size}\;N$15:$T\leftarrow \mathrm{BuildKDTree}(H_{s})$16:**for** i←1toN **do**17:      $h_{i}\leftarrow \mathrm{NearestNeighbor}\left(p_{\mathrm{norm},i},T\right)$18:      $d_{i}\leftarrow \mathrm{DistanceAlongCurve}\left(H_{s},h_{i}\right)$19:      $D_{\mathrm{Hilbert}}\left[i\right]\leftarrow d_{i}$20:**end for**21:$P_{\mathrm{sorted}}\leftarrow \mathrm{Sort}\left(P,D_{\mathrm{Hilbert}}\right)$
22:
23:**Step 2:** Inverse Sorting Vector Computing for LDS24:a←arrayofsizeN25:**for** i←1toN **do**26:      a[i]←(i·e)mod127:**end for**28:$a^{\prime }\leftarrow \mathrm{Sort}\left(a\right)$29:r←arrayofsizeN30:**for** i←1toN **do**31:      Find *k* such that $a^{\prime }\left[k\right]=a\left[i\right]$32:      r[i]←k33:**end for**
34:
35:**Step 3:** Reordering Point Cloud with LDS Inverse Sorting Vector36:$P_{\mathrm{reordered}}\leftarrow \left(\right)$37:**for** k←1toN **do**38:      $P_{\mathrm{reordered}}\leftarrow P_{\mathrm{reordered}}\cup \left(P_{\mathrm{sorted}}\left[r\left[k\right]\right]\right)$39:**end for**
40:
41:**Step 4:** Select Subsequence42:$P^{\prime }\leftarrow P_{\mathrm{reordered}}\left[1\ldots M\right]$43:**return** $P^{\prime }$


During the initial sorting phase (Step 1), the vertices of the *s*-order Hilbert curve $H_{s}$ can be pre-computed and stored for commonly used orders *s* and dimensions *d* to accelerate the process.

When projecting points onto the Hilbert curve, a KD-Tree is constructed from the vertices of $H_{s}$. This data structure is then used to efficiently find the nearest point $h_{i}$ on the Hilbert curve for each normalized point $p_{\mathrm{norm},i}$. The Hilbert index $d_{i}$ is the distance along the curve from its starting point to $h_{i}$.

In Step 2, for generating LDS, Euler’s number (e≈2.71828) is used as the transcendental number due to its simplicity and good practical performance in generating sequences with favorable distribution properties.

The inverse sorting vector r computed in Step 2 essentially maps the original LDS indices to their sorted ranks. When this vector is used to reorder the Hilbert-sorted point cloud in Step 3, it effectively shuffles the spatially coherent sequence $P_{\mathrm{sorted}}$ in a quasi-random manner, breaking up local clusters while attempting to maintain a good global distribution for any sub-sequence taken from the beginning.

### 3.2. Theoretical Foundation and Analysis

The superior performance of our LDS-Hilbert method derives from the complementary mathematical properties of Low-Discrepancy Sequences and Hilbert curve ordering, which together create a synergistic approach to point cloud down-sampling.

#### 3.2.1. Hilbert Curve Properties

Step 1 leverages the Hilbert curve’s locality-preserving property, which has been rigorously established by Moon et al. [46] and applied successfully to point cloud processing by Chen et al. [49] and Pandhare [48]. The Hilbert curve mapping $H:\left[0,1\right]\to {\left[0,1\right]}^{d}$ preserves locality in a precise mathematical sense: for any δ>0, there exists ϵ>0 such that if |s−t|<ϵ, then |H(s)−H(t)|<δ.

As shown by Gotsman [50], the Hilbert curve minimizes the average locality distortion among all space-filling curves, making it optimal for preserving neighboring relationships. This mapping ensures that points spatially close in the original 3D space remain close in the 1D sequence, preserving neighborhood relationships through the transformation.

Our implementation uses the bit-manipulation approach introduced by Skilling [51] rather than recursive geometric constructions, making it computationally efficient with O(s·d) complexity per point. This efficiency is critical for processing large point clouds with millions of points.

#### 3.2.2. Low-Discrepancy Sequence Properties

Low-Discrepancy Sequences inherently excel at achieving uniform coverage of sampling domains through their fundamental property: minimizing the deviation between the empirical distribution of points and the uniform distribution. This deviation, formally quantified as discrepancy, is mathematically expressed as:

$$D_{N}\left(P\right)=\underset{B\in B}{\sup}\left|\frac{\left|P\cap B\right|}{N}-\lambda \left(B\right)\right|$$

where *P* is a point set with *N* points, B is a family of measurable subsets, and λ(B) is the Lebesgue measure of set *B*. LDS construction ensures this discrepancy decreases at a rate of $O({\left(\log N\right)}^{d}/N)$ compared to the $O(1/\sqrt{N})$ rate of pseudo-random sequences, providing theoretically superior domain coverage with fewer samples [28,34].

The use of Euler’s number *e* as the base for our LDS follows the well-established additive recurrence relation method for generating Low-Discrepancy Sequences [28,52,53], which guarantees optimal uniformity of the sampling distribution.

#### 3.2.3. Synergistic Combination

The synergy in LDS-Hilbert arises from addressing the distinct challenges of achieving both local feature preservation and global sampling uniformity. While Hilbert ordering (Section 3.2.1) provides a spatially coherent 1D path, naive subset selection from this path can be biased and does not inherently guarantee optimal discrepancy for the selected *M* points. Conversely, directly applying LDS principles (Section 3.2.2) to an unordered point cloud might disregard the intrinsic manifold structure and local density variations of the data.

Our method leverages the Hilbert curve to first establish this crucial 1D spatial coherence. Then, the LDS-based reordering ensures that points are selected from all segments along this coherent path in a quasi-random, uniformly distributed manner. This two-stage process thereby samples the local spatial relationships (preserved by Hilbert) with global representativeness (ensured by LDS). This integrated strategy fundamentally distinguishes our approach from prior methods that might employ SFCs primarily for voxel flattening and 2D processing [8] or use LDS with simpler, less locality-aware initial orderings, such as the PCA-based ordering explored by Xu et al. [7]. The unique contribution of the Hilbert curve component within our synergistic framework is empirically evaluated by comparing its performance against LDS-PCA [7] in our experiments.

#### 3.2.4. Starting Index Selection and Contiguous Sampling

Our decision to select the first M points (setting s=1) from the LDS-reordered sequence is theoretically justified by the mathematical properties of Low-Discrepancy Sequences. After the reordering in Step 3, the point cloud $P_{\mathrm{reordered}}$ already embodies the optimal distribution properties of the LDS, effectively distributing sample points uniformly across the entire Hilbert-ordered space. Due to this property, any contiguous block of M points from this sequence will maintain representative coverage of the original point cloud’s domain.

Our decision to select the first contiguous block of *M* points is not only straightforward but also theoretically grounded. Since the Hilbert curve maps spatially proximate points to nearby positions in the 1D sequence, selecting the first contiguous block preserves feature clusters and geometric relationships that might otherwise be fragmented if points were selected at fixed intervals or based on rank thresholds. Therefore, in our implementation, we use a deterministic approach by consistently setting the starting index to s=1 across all experiments.

#### 3.2.5. Computation and Storage Complexity

Computationally, the primary costs of the LDS-Hilbert algorithm stem from the sorting steps and the Hilbert curve mapping. Sorting the original points by their Hilbert indices and sorting the LDS sequence to compute the inverse sorting vector both have a typical time complexity of O(NlogN), where *N* is the number of input points.

Regarding storage for the Hilbert curve mapping, the strategy involves pre-computing vertices for commonly used orders *s*. A *d*-dimensional Hilbert curve of order *s* has $N_{H}={\left(2^{d}\right)}^{s}$ vertices. Storing these $N_{H}$ vertices, each with *d* coordinates, requires $O(N_{H}\cdot d)$ space, and loading them from disk is an efficient I/O operation. The order *s* is typically chosen such that the $N_{H}\approx O\left(N\right)$ to provide sufficient granularity for mapping. Consequently, the storage for pre-computed vertices scales as O(N·d).

The computational aspect of Hilbert curve mapping, using these $N_{H}$ pre-computed vertices, involves building a spatial indexing structure (e.g., a KD-Tree) on them, which takes $O(N_{H}\log N_{H})$ time. Since $N_{H}\approx O\left(N\right)$, this step is O(NlogN). Subsequently, for each of the *N* input points, querying this structure to find the nearest curve vertex and projecting the point to the relevant curve segments takes approximately $O(\log N_{H}+d)$ per point, leading to a total of O(N(logN+d)) for all input points.

Thus, the overall theoretical time complexity, combining sorting and the Hilbert mapping with pre-computed vertices, is dominated by these steps, generally scaling as O(NlogN+N·d). This makes the method efficient relative to approaches with quadratic or higher complexity, especially for large datasets.

#### 3.2.6. Summary of Advantages

This theoretical foundation explains our method’s empirical superiority:

1. Global distribution preservation: By sampling uniformly across the Hilbert-ordered sequence rather than in 3D space directly, our method maintains the original density variations that often encode important structural information.
2. Local feature preservation: The locality preservation property of the Hilbert curve ensures that important local features are not arbitrarily split across distant indices, making them less susceptible to being eliminated during the sampling process.
3. Deterministic coverage guarantees: Unlike stochastic methods, our approach provides mathematical guarantees on sampling coverage through the discrepancy bounds of LDS, ensuring no significant regions of the point cloud are overlooked.

We conduct four experiments to test this theoretical analysis across diverse geometry types and metrics.

## 4. Materials and Experiments

### 4.1. Digital Twin Categorization and Fidelity Measurement

**Figure 1:**
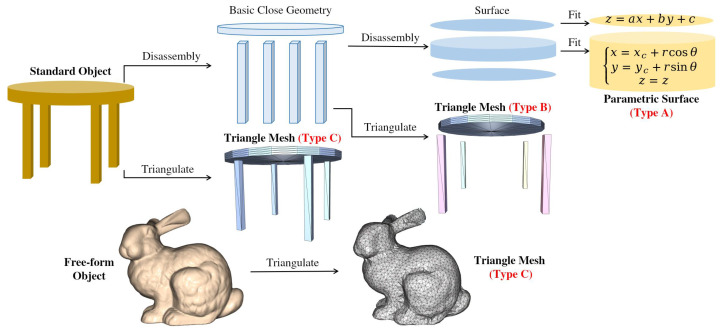
Digital twin categorization based on object complexity and reconstruction approach.

Digital twins represent virtual replicas of physical objects, and their construction methodology significantly impacts their fidelity and utility. As illustrated in Figure 1, we categorize digital twins ($M^{\prime }$) created from down-sampled point clouds ($P^{\prime }$) into three distinct types based on the original object’s geometry ($M_{\mathrm{true}}$) and the reconstruction approach ($g:P^{\prime }\to M^{\prime }$).

For objects with predominantly primitive or standard surface geometry, two reconstruction approaches are viable. Type A digital twins employ parametric representation, where the model is defined by fitted geometric primitives (planes, spheres, cylinders, etc.) or standard parametric surfaces. This approach involves segmenting the down-sampled point cloud and applying least-squares methods to estimate parameters ($w_{\mathrm{est}}$) that define these geometric entities. As shown in Figure 1, surfaces like planes and cylinders can be effectively represented by their mathematical equations, yielding compact yet precise digital representations.

Alternatively, Type B digital twins utilize mesh-based representation for parameterizable objects. Although the underlying geometry could be described parametrically, this approach directly applies surface reconstruction algorithms (such as Ball Pivoting, Poisson Surface Reconstruction, or Alpha Shapes) to the down-sampled point cloud. The resulting triangle mesh provides visual fidelity while maintaining a direct connection to the original geometric properties. Figure 1 demonstrates how a standard object like a table can be disassembled into basic closed geometries (cylindrical top and rectangular legs), which can then be individually triangulated to form a complete mesh model.

For objects with free-form or organic geometry (such as the bunny model shown in Figure 1), Type C digital twins are inherently represented as triangle meshes since their complexity makes parametric fitting impractical. This approach applies surface reconstruction algorithms directly to the down-sampled point cloud to generate a mesh that captures the intricate shapes and features of complex objects. The figure clearly illustrates how triangulation transforms the smooth, organic bunny shape into a detailed mesh representation capable of preserving its distinctive features.

The fidelity assessment for each digital twin type requires specialized metrics tailored to their representation. For Type A twins, we evaluate parameter accuracy, while Types B and C necessitate geometric comparison measures between reconstructed surfaces and ground truth data. This systematic categorization forms the foundation for our experimental evaluation of different down-sampling methods and their impact on digital twin fidelity.

The assessment of fidelity, denoted as $D(M^{\prime },M_{\mathrm{true}})$, employs metrics specifically chosen based on the type of digital twin being evaluated.

For Type A digital twins, where the reconstructed model $M^{\prime }$ is represented by a fitted surface defined by a parameter vector w, fidelity is measured by the discrepancy between the estimated parameters $w_{\mathrm{est}}$ and the ground truth parameters $w_{\mathrm{true}}$. A robust method for quantifying this difference is the L1 norm, chosen for its resilience to outliers. This metric is calculated as $D_{L1}=∥w_{\mathrm{est}}-w_{\mathrm{true}}{∥}_{1}=\sum_{i=1}^{k}\left|w_{\mathrm{est},i}-w_{\mathrm{true},i}\right|$, where *k* represents the number of parameters in the vector w.

When the digital twin $M^{\prime }$ is represented as a mesh (applicable to both Type B and Type C), the fidelity assessment relies on metrics that quantify the geometric discrepancy between the reconstructed representation and the ground truth. This involves comparing either the down-sampled point cloud $P^{\prime }$ or the reconstructed mesh $M^{\prime }$ against the ground truth mesh $M_{\mathrm{true}}$ or the ground truth point cloud $P_{\mathrm{true}}$. Several geometric metrics are utilized for this purpose.

One such metric is the Point-to-Mesh Distance ($D_{p2m}$). This asymmetric measure calculates the average distance from each point $p_{i}^{\prime }$ in the down-sampled point cloud $P^{\prime }={\left\{p_{i}^{\prime }\right\}}_{i=1}^{M}$ to its nearest corresponding point $p_{\mathrm{true}}$ on the continuous surface $S(M_{\mathrm{true}})$ of the ground truth mesh. It effectively quantifies how closely the sampled points conform to the actual surface, providing insight into reconstruction accuracy from the sample’s perspective. The formulation is $D_{p2m}\left(P^{\prime },M_{\mathrm{true}}\right)=\frac{1}{M}\sum_{i=1}^{M}\min_{p_{\mathrm{true}}\in S\left(M_{\mathrm{true}}\right)}{∥p_{i}^{\prime }-p_{\mathrm{true}}∥}_{2}$, and it is often computed using tools like Metro [54].

Another key metric is the Chamfer Distance ($D_{\mathrm{CD}}$), a symmetric measure evaluating the similarity between two point sets, $P^{\prime }$ and $P_{\mathrm{true}}$. It provides a global assessment of alignment by summing the average distance from each point in one set to its nearest neighbor in the other set, and vice versa. Using non-squared distances, the formulation is $D_{\mathrm{CD}}\left(P^{\prime },P_{\mathrm{true}}\right)=\frac{1}{M}\sum_{p^{\prime }\in P^{\prime }}\min_{p_{\mathrm{true}}\in P_{\mathrm{true}}}∥p^{\prime }-p_{\mathrm{true}}{∥}_{2}+\frac{1}{N}\sum_{p_{\mathrm{true}}\in P_{\mathrm{true}}}\min_{p^{\prime }\in P^{\prime }}{∥p_{\mathrm{true}}-p^{\prime }∥}_{2}$. Although the original concept is older [55], it has been widely adopted, particularly in deep learning contexts [56], often using the sum of the average squared distances in implementations.

To address the sensitivity of the standard Hausdorff Distance [57] to outliers, the Percentile Hausdorff Distance ($D_{H,p}$) is employed. This more robust metric calculates a specific percentile (e.g., 95th) of the nearest neighbor distances rather than the maximum. We consider its two directional components separately, as discussed in metric evaluation surveys [58]. The sample-to-ground truth component ($D_{H,95,\;s2g}$) measures the 95th percentile of distances from points in $P^{\prime }$ to their nearest neighbors in $P_{\mathrm{true}}$, indicating how well $P^{\prime }$ is covered by the true surface while tolerating some outliers in $P^{\prime }$. Conversely, the ground truth-to-sample component ($D_{H,95,\;g2s}$) measures the 95th percentile of distances from points in $P_{\mathrm{true}}$ to their nearest neighbors in $P^{\prime }$, indicating how well the sample $P^{\prime }$ covers the true surface, tolerating some unrepresented points in $P_{\mathrm{true}}$. Letting $d_{i\to \mathrm{true}}=\min_{p_{\mathrm{true}}\in P_{\mathrm{true}}}{∥p_{i}^{\prime }-p_{\mathrm{true}}∥}_{2}$ and $d_{j\to \mathrm{sample}}=\min_{p^{\prime }\in P^{\prime }}{∥p_{\mathrm{true},j}-p^{\prime }∥}_{2}$, these are formulated as $D_{H,95,\;s2g}\left(P^{\prime },P_{\mathrm{true}}\right)={\mathrm{Percentile}}_{95\%}\left({\left\{d_{i\to \mathrm{true}}\right\}}_{i=1}^{M}\right)$ and $D_{H,95,\;g2s}\left(P^{\prime },P_{\mathrm{true}}\right)={\mathrm{Percentile}}_{95\%}\left({\left\{d_{j\to \mathrm{sample}}\right\}}_{j=1}^{N}\right)$.

Finally, the Viewpoint Feature Histogram (VFH) Distance ($D_{\mathrm{VFH}}$) assesses similarity based on global shape and viewpoint characteristics. VFH itself is a global descriptor for 3D point clouds, encoding geometric information derived from surface normals and viewpoint directions [59,60]. The $D_{\mathrm{VFH}}$ is not a direct spatial distance between points but rather a distance computed between the VFH descriptor histograms ($h_{\mathrm{VFH}}$) of the reconstructed model/point cloud ($P^{\prime }$ or $M^{\prime }$) and the ground truth model/point cloud ($P_{\mathrm{true}}$ or $M_{\mathrm{true}}$). A smaller distance, typically calculated using the L2 norm $d_{\mathrm{hist}}\left(h_{1},h_{2}\right)={∥h_{1}-h_{2}∥}_{2}$, indicates greater similarity in the overall shape and viewpoint properties captured by the histograms: $D_{\mathrm{VFH}}\left(P^{\prime },P_{\mathrm{true}}\right)=d_{\mathrm{hist}}\left(h_{\mathrm{VFH}}\left(P^{\prime }\right),h_{\mathrm{VFH}}\left(P_{\mathrm{true}}\right)\right)$.

### 4.2. Experimental Design

To rigorously evaluate the efficacy of the proposed Low-Discrepancy Sequences-driven down-sampling method using Hilbert curve ordering (LDS-Hilbert), we designed a series of four comprehensive experiments. These experiments assess the method’s performance across a spectrum of object complexities and digital twin reconstruction tasks, comparing it against three widely recognized benchmark down-sampling techniques: Simple Random Sampling (SRS), Farthest Point Sampling (FPS), and Voxel Grid Filtering (Voxel). Furthermore, to isolate the contribution of the Hilbert curve ordering component within our proposed framework, we include an ablation study in each experiment by evaluating a variant of our method that replaces the Hilbert curve sorting step with sorting based on the first principal component obtained via Principal Component Analysis (PCA), referred to as LDS-PCA.

All experiments were implemented in Python 3.8 on a laptop with an Intel Core i7-11800H CPU (Intel, Santa Clara, CA, USA). Core point cloud and mesh operations utilized Open3D [61] and NumPy [62]. VFH computation leveraged the Python interface to the Point Cloud Library (PCL) [14]. Farthest Point Sampling (FPS) was implemented via the fpsample library [63], and Hilbert curve generation used the numpy-Hilbert-curve package [64] which relies on Skilling’s work [51]. Additional libraries, such as Scikit-learn [65] and SciPy [66], were employed for specific tasks like PCA and parametric fitting.

To provide a direct comparison of LDS-Hilbert against the other methods (SRS, FPS, Voxel, LDS-PCA), we introduce a **Performance Ratio** for each fidelity metric and each object. Let $D_{k,j}\left(\mathrm{Method}\right)$ denote the calculated value of the *k*-th fidelity metric (i.e., $D_{L1}$, $D_{p2m}$, $D_{\mathrm{CD}}$, $D_{H,95,\;s2g}$, $D_{H,95,\;g2s}$, $D_{\mathrm{VFH}}$) for the *j*-th object using a specific down-sampling method. For a given comparison method Comp (where Comp ∈ SRS, FPS, Voxel, LDS-PCA), the Performance Ratio relative to LDS-Hilbert is calculated as:

$$R_{k,j}\left(\mathrm{Comp}\;\mathrm{vs}.\;\mathrm{LDS}\text{-}\mathrm{Hilbert}\right)=\frac{D_{k,j}\left(\mathrm{Comp}\right)}{D_{k,j}\left(\mathrm{LDS}\text{-}\mathrm{Hilbert}\right)}$$

Since lower values indicate higher fidelity for all metrics used in these experiments, a ratio $R_{k,j}>1$ signifies that the LDS-Hilbert method achieved a better (lower) score and thus outperformed the comparison method ‘Comp’ for that specific metric *k* and object *j*.

To summarize the overall performance within each experiment, we calculate the percentage of objects for which LDS-Hilbert outperforms each comparison method on a given metric. Let $N_{\mathrm{obj}}$ be the total number of objects in an experiment. For a specific metric *k* and comparison method Comp, we count the number of objects $N_{\mathrm{win}}$, where $R_{k,j}\left(\mathrm{Comp}\;\mathrm{vs}.\;\mathrm{LDS}\text{-}\mathrm{Hilbert}\right)>1$. The **Win Percentage** is then:

$$\mathrm{Win}\;{\mathrm{Percentage}}_{k}\left(\mathrm{LDS}\text{-}H\;\mathrm{vs}.\;\mathrm{Comp}\right)=\frac{N_{\mathrm{win}}}{N_{\mathrm{obj}}}\times 100\%$$

A Win Percentage greater than 50% indicates that LDS-Hilbert outperformed the comparison method ‘Comp’ on that specific metric for the majority of objects within that experiment. This systematic evaluation across diverse datasets, reconstruction tasks, and metrics aims to provide robust evidence regarding the efficacy of the proposed LDS-Hilbert down-sampling method in enhancing digital twin fidelity.

### 4.3. Experiment 1: Parametric Surface Fidelity

This experiment evaluated the capability of various down-sampling methods (LDS-Hilbert, LDS-PCA, SRS, FPS, Voxel) to retain geometric information essential for accurately recovering the parameters of simple, known shapes (Type A Digital Twin). The dataset consisted of synthetic point clouds generated by sampling points uniformly from seven distinct analytical surfaces: plane, sphere, ellipsoid, torus, cylinder, cone, and paraboloid, each defined by specific ground truth parameters (e.g., sphere radius = 1, ellipsoid semi-axes = 3,2,1; see Appendix B for complete details). Points were sampled within defined parameter ranges for each surface type. To simulate measurement error, controlled Gaussian noise (σ=0.05 relative to object scale) was added independently to the coordinates of each point.

Point clouds were generated at three sizes (*N* = 9180, *N* = 37,240 and *N* = 95,760), resulting in 21 unique synthetic objects. Each down-sampling method reduced the point clouds to 10% and 20% of their original size (ratio = 0.10 and ratio = 0.20). The resulting down-sampled point cloud $P^{\prime }$ was then used to fit the corresponding known parametric surface equation using a least-squares optimization method (see Appendix B for details). Performance was measured using the L1 norm of the parameter difference ($D_{L1}$) between the estimated parameters $w_{\mathrm{est}}$ derived from $P^{\prime }$ and the known ground truth parameters $w_{\mathrm{true}}$.

**Figure 2:**
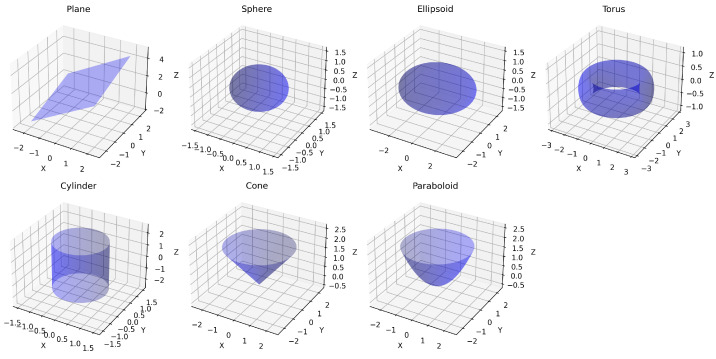
The seven parametric surfaces used in Experiment 1.

Figure 2 illustrates the seven parametric surfaces used in our evaluation. These surfaces represent a diverse set of geometric primitives commonly encountered in CAD models and industrial environments, ranging from simple planar surfaces to more complex curved geometries like tori and paraboloids. This diversity enables comprehensive evaluation of each down-sampling method’s ability to preserve parametric characteristics across different surface types.

#### 4.3.1. Quantitative Results

Table 1 and Table 2, respectively, present the Performance Ratio and Win Percentage metrics for the L1 parameter distance. The Performance Ratio quantifies the relative error of each method compared to LDS-Hilbert, with values greater than 1.0 indicating LDS-Hilbert’s superior performance. The Win Percentage shows the proportion of test cases where LDS-Hilbert outperformed each comparison method. To analyze the effects of point cloud size (*N*) and the down-sampling ratio on the results, the data are grouped by these factors, with each value representing the average across all surface types.

#### 4.3.2. Key Findings

Three critical patterns emerge from this analysis:

1. **Scale-dependent advantage of LDS-Hilbert:** The performance benefit of LDS-Hilbert becomes more pronounced as point cloud size (*N*) increases. At N=9180, its advantage over methods like SRS or LDS-PCA is marginal (e.g., ratios of 0.97 vs. SRS and 0.84 vs. LDS-PCA at 20% sampling). However, at *N* = 95,760, LDS-Hilbert significantly outperforms them (e.g., ratios up to 1.29 vs. SRS and 1.18 vs. LDS-PCA, with 100% win rates against both in some cases). This scale dependency likely arises because larger *N* allows the Hilbert curve (with a correspondingly higher order *s*) to provide a more granular, locality-preserving 1D mapping, and the LDS reordering can more effectively distribute selection ranks across this longer, more detailed sequence.
2. **Consistent superiority over structured methods:** Regardless of point cloud size, LDS-Hilbert significantly outperforms both the FPS and Voxel methods across nearly all test conditions. Performance ratios consistently remain above 1.24 and reach as high as 3.07 for FPS at *N* = 95,760 with 10% down-sampling. Table 2 demonstrates that for the largest point clouds, LDS-Hilbert achieves a 100% win rate against these methods, indicating universal superiority for parameter recovery tasks at scale.
3. **Evolving role of Hilbert vs. PCA ordering:** The comparison with LDS-PCA (using Principal Component Analysis for initial ordering) shows that at smaller scales (N=9180), LDS-PCA can be competitive to LDS-Hilbert. This suggests PCA’s global variance capture might suffice for simpler distributions. However, at *N* = 95,760, LDS-Hilbert consistently surpasses LDS-PCA. This indicates that as point density increases, the Hilbert curve’s superior ability to preserve local neighborhood relationships across the entire manifold becomes more critical for accurate fitting than the global structure captured by PCA.

Notably, while the relative performance ranking of the methods remains largely consistent across different down-sampling ratios (10% vs. 20%), LDS-Hilbert demonstrates particular robustness and often a greater magnitude of performance advantage at the more aggressive 10% ratio. This highlights its effectiveness precisely when substantial data reduction is required, suggesting its advantages become more pronounced under stricter down-sampling constraints.

See Section 4.7 for a paired *t*-test of the significance level used to measure the superiority of LDS-Hilbert.

#### 4.3.3. Implications

These results provide strong evidence that the LDS-Hilbert method significantly improves parametric surface reconstruction fidelity compared to traditional methods. Its advantages become most apparent when handling large point clouds requiring substantial down-sampling—precisely the scenario most relevant to practical digital twin applications where computational efficiency must be balanced with model accuracy.

### 4.4. Experiment 2: Mesh Reconstruction from Closed Prismatic Geometry

This experiment investigated the performance of down-sampling methods in reconstructing mesh-based digital twins (Type B) from point clouds representing regular, closed geometric shapes. These shapes featured both planar and curved faces, as well as sharp edges. The dataset was generated from 15 distinct mesh-based geometric models, including shapes like spheres, cylinders, various prisms (triangular, cube, rectangular, pentagonal, hexagonal), structural beams (L, T, U, H types), cones, tori, and basic polyhedra (tetrahedron, octahedron), with precisely defined dimensions based on a scale factor (see Appendix C for more details). Before sampling, each mesh model underwent a random rotation. Points were then sampled relatively uniformly from the surface of each rotated mesh using the Poisson disk sampling method, generating clouds at three sizes (*N* = 9180, *N* = 37,240 and *N* = 95,760) for a total of 45 objects. Gaussian noise with a standard deviation of 1 cm (assuming meter scale for the objects) was added to the coordinates of each sampled point to simulate LiDAR scanning errors. Each down-sampling method reduced the input cloud P to 10% (ratio = 0.10), as Experiment 1 demonstrated that the ratio had minimal impact on the relative performance of methods.

The resulting down-sampled cloud $P^{\prime }$ was then used as input for surface reconstruction via the Ball Pivoting algorithm (*g*) to create a mesh digital twin $M^{\prime }$ (see Appendix D for Ball Pivoting details).

Fidelity was evaluated by comparing $P^{\prime }$ against the ground truth mesh $M_{\mathrm{true}}$ and the ground truth point cloud $P_{\mathrm{true}}$ (vertices of the original mesh before noise) using a suite of geometric metrics: Point-to-Mesh Distance ($D_{p2m}$), Chamfer Distance ($D_{\mathrm{CD}}$), the 95th Percentile Hausdorff Distance components ($D_{H95,s2g}$ and $D_{H95,g2s}$), and Viewpoint Feature Histogram Distance ($D_{\mathrm{VFH}}$).

#### 4.4.1. Visual Assessment

**Figure 3:**
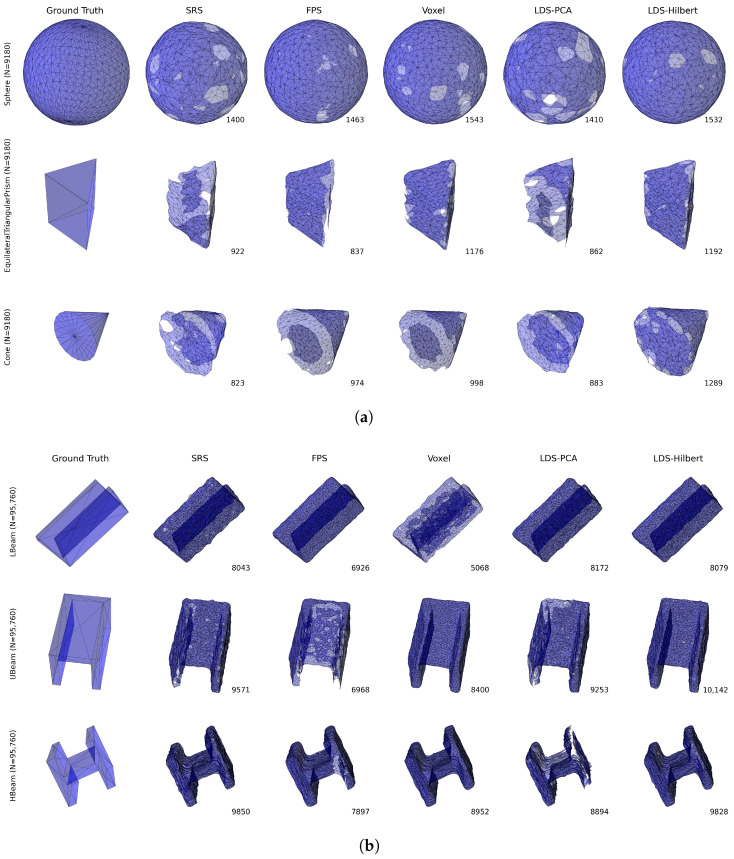
Visual comparison of surface reconstruction results for: (**a**) Sphere, triangular prism, and cone; (**b**) Structural beams (L, U, and H profiles).

Figure 3a,b provide visual comparisons of the reconstruction results across different down-sampling methods. Figure 3a shows the results for simpler geometries (sphere, triangular prism, and cone), while Figure 3b presents more complex structural shapes (L-beam, U-beam, and H-beam).

Visual inspection of Figure 3a reveals that for the sphere (N=9180), all methods produce reasonably complete reconstructions, but SRS, FPS, and to some extent LDS-PCA, create noticeable holes or irregularities on the surface. The LDS-Hilbert method produces a more complete and uniform sphere reconstruction with a high face count (1532), comparable to the Voxel method (1543).

For shapes with sharp features, such as the triangular prism (N=9180) and cone (N=9180), the differences become more pronounced. SRS produces highly irregular reconstructions with significant gaps, while FPS creates smoother but sometimes incomplete reconstructions. The LDS-Hilbert method achieves the highest face counts (1192 for the triangular prism and 1289 for the cone), indicating more complete mesh reconstruction that better preserves the sharp geometric features.

Figure 3b demonstrates even more significant differences with complex structural shapes. The SRS method tends to produce irregular meshes with inconsistent densities. While FPS generates smoother surfaces, it often fails to maintain proper thickness in the thin sections of these complex shapes. The Voxel method struggles with the interior corners of the U-beam and H-beam. In contrast, the LDS-Hilbert method consistently achieves the highest face counts (8079 for L-beam, 10,142 for U-beam, and 9828 for H-beam) and visually preserves both the overall structure and fine details of these challenging geometries.

#### 4.4.2. Quantitative Results

Table 3 and Table 4 present the Performance Ratio and Win Percentage metrics for each evaluation criterion, grouped by point cloud size.

The Performance Ratio quantifies the relative error of each comparison method to LDS-Hilbert (values greater than 1.0 indicate LDS-Hilbert’s superior performance), while the Win Percentage shows the proportion of test cases where LDS-Hilbert outperformed each method.

The quantitative metrics align with the visual observations from Figure 3a,b, demonstrating several clear patterns across different metrics and point cloud sizes.

In addition, we also provide computational time comparisons in Appendix E to validate the efficiency of our proposed method.

#### 4.4.3. Key Findings

1. **Progressive advantage with scale**: For nearly all metrics, LDS-Hilbert’s advantage increases with point cloud size. This is particularly evident in the Point-to-Mesh Distance ($D_{p2m}$) metric, where the Performance Ratio against FPS increases from 1.39 at N=9180 to 1.64 at *N* = 95,760, and against Voxel from 0.89 at N=9180 to 1.36 at *N* = 95,760. Similarly, the Win Percentage against Voxel for this metric rises dramatically from 6.67% at N=9180 to 100% at *N* = 95,760.
2. **Superior morphological fidelity**: The Viewpoint Feature Histogram (VFH) Distance shows the most dramatic improvement, with Performance Ratios at *N* = 95,760 reaching 1.96, 1.96, 1.85, and 1.86 against SRS, FPS, Voxel, and LDS-PCA, respectively. This substantial difference (nearly twice as good) indicates that LDS-Hilbert preserves the overall geometric characteristics and surface morphology significantly better than all comparison methods, with 100% win rates against SRS, FPS, and LDS-PCA at the largest scale.
3. **Trade-off in coverage metrics**: For the Hausdorff Distance from ground truth to sample ($D_{H,95,g2s}$), the FPS and Voxel methods achieved lower error rates than LDS-Hilbert at smaller scales, with Performance Ratios of 0.88 and 0.87, respectively, at N=9180. This reflects these methods’ design focus on ensuring uniform coverage. However, this advantage diminishes at larger scales, with ratios increasing to 1.04 for FPS at *N* = 95,760, suggesting that LDS-Hilbert’s balanced approach becomes increasingly effective as complexity grows.
4. **Comprehensive distance metrics**: The Chamfer Distance ($D_{\mathrm{CD}}$), which considers both how well sampled points represent the surface and how well the surface is covered by sampled points, shows LDS-Hilbert outperforming the comparison methods across most conditions. At *N* = 95,760, LDS-Hilbert achieves a 100% win rate against all comparison methods, demonstrating its balanced approach to addressing both objectives.
5. **Value of Hilbert ordering**: The comparison with LDS-PCA isolates the contribution of the Hilbert curve ordering. While LDS-PCA performs relatively well compared to other methods, LDS-Hilbert consistently outperforms it at the largest scale (*N* = 95,760) across all metrics, with Performance Ratios of 1.00–1.86 and Win Percentages of 40–100%. This confirms that the Hilbert curve’s spatial coherence properties specifically enhance reconstruction quality.

The statistical significance of these results is analyzed in Section 4.7.

#### 4.4.4. Implications and Synthesis

Combining the visual assessment with the quantitative results yields several important insights about LDS-Hilbert’s performance in mesh reconstruction tasks:

1. LDS-Hilbert provides consistent, low-error results in surface reconstruction, particularly for large point clouds requiring significant down-sampling. The consistently higher face counts across diverse geometries indicate more complete reconstructions that better preserve the original shape characteristics.
2. While SRS produces down-sampled point clouds with moderate numerical differences from ground truth (Performance Ratios of 1.00–1.21 for most metrics), its high randomness and uneven distribution result in poor visual quality, evidenced by numerous holes and irregular surfaces in Figure 3a,b.
3. The FPS and Voxel methods create visually smoother reconstructions for simpler convex geometries but struggle with shapes having sharp features (triangular prisms, cones) and complex structures with concave regions (U-beams, H-beams). These limitations are reflected in their higher error rates at larger scales.
4. The most significant advantage of LDS-Hilbert appears in preserving the overall shape characteristics and surface morphology, as demonstrated by the substantial improvements in the VFH Distance at *N* = 95,760. This suggests that the method is particularly valuable for applications where faithful reproduction of an object’s appearance and geometric features is critical.

These findings demonstrate that LDS-Hilbert offers substantial advantages for mesh reconstruction from closed prismatic geometries, with benefits that become increasingly pronounced as point cloud size and shape complexity increase—precisely the conditions encountered in practical digital twin applications.

### 4.5. Experiment 3: Mesh Reconstruction from Diverse CAD Models (ModelNet40)

This experiment focused on evaluating the generalization ability of the down-sampling methods across a broad spectrum of complex object shapes (Type C), often consisting of multiple components. The experiment utilized 40 CAD models selected from the ModelNet40 dataset, one from each class. A ground truth watertight mesh and corresponding point cloud were established for each model, and Gaussian noise was added to create the input point clouds. Following the standard procedure, each method down-sampled the input clouds to a 20% ratio. The resulting point clouds were then reconstructed into Type C Mesh digital twins using the Ball Pivoting algorithm. Performance was quantitatively evaluated using the same suite of geometric metrics as in Experiment 2, comparing the down-sampled cloud against the ground truth mesh and the ground truth point cloud.

#### 4.5.1. Visual Assessment

**Figure 4:**
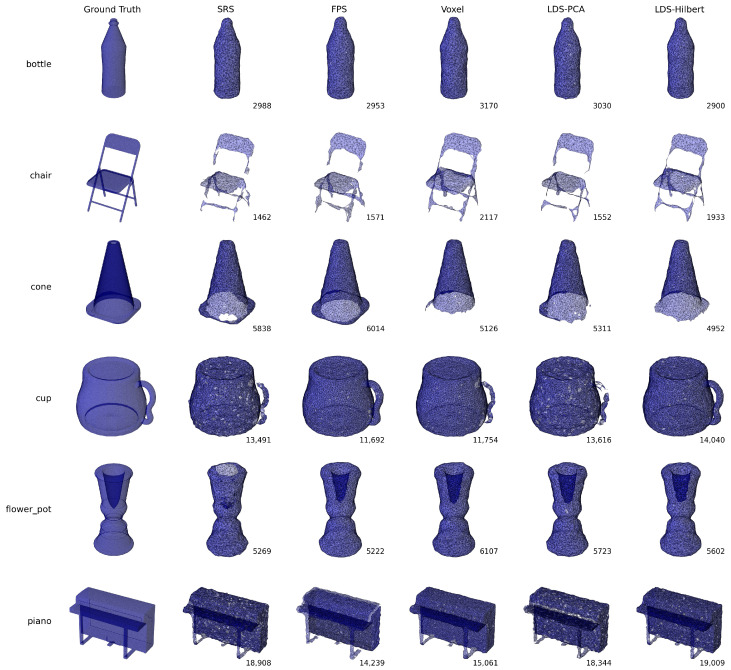
Visual comparison of surface reconstruction results for selected ModelNet40 objects.

Figure 4 provides a visual comparison of reconstruction results across different down-sampling methods for six representative objects: bottle, chair, cone, cup, flower pot, and piano. The ground truth is shown in the leftmost column, with reconstructed meshes from each method in subsequent columns. The numbers indicate the face count of each reconstructed mesh.

Visual examination reveals clear differences in reconstruction quality across the methods. Similar to our observations in Experiment 2, the FPS and Voxel methods tend to produce smoother surfaces but frequently fail at modeling concave regions and sharp features. This is particularly evident in the chair model, where the FPS method (1571 faces) struggles to properly reconstruct the seat and back connection, while the LDS-Hilbert method (1933 faces) preserves more structural details and achieves a higher face count.

The SRS and LDS-PCA methods consistently generate reconstructions with numerous surface irregularities. For example, in the bottle model, both methods show noticeable bumps and inconsistencies along what should be smooth surfaces. While SRS occasionally achieves high face counts (as seen in the piano model with 18,908 faces), the visual quality of these reconstructions remains problematic with multiple gaps and irregular surfaces.

The LDS-Hilbert method demonstrates superior performance in preserving both smooth regions and detailed structures. This is clearly visible in the cup model, where LDS-Hilbert (14,040 faces) captures the hollow interior and handle with greater fidelity than other methods. For objects with more complex geometry, such as the piano, LDS-Hilbert (19,909 faces) achieves the highest face count and visually appears to best preserve the original shape, including the keyboard area and supporting structure.

#### 4.5.2. Quantitative Results

Due to the extreme complexity of one model (Keyboard_0001), all methods failed to produce acceptable reconstructions, so this model was excluded from the quantitative analysis. For the remaining 39 models, Table 5 and Table 6 present the Performance Ratio and Win Percentage metrics for each evaluation criterion.

The Performance Ratio (Table 5) quantifies the relative error of each comparison method to LDS-Hilbert, with values greater than 1.0 indicating LDS-Hilbert’s superior performance. The Win Percentage (Table 6) shows the proportion of test cases where LDS-Hilbert outperformed each comparison method. These metrics were calculated across all five geometric distance measures: Point-to-Mesh Distance ($D_{p2m}$), Chamfer Distance ($D_{\mathrm{CD}}$), both components of the 95th percentile Hausdorff Distance ($D_{H,95,s2g}$ and $D_{H,95,g2s}$), and the VFH Distance ($D_{\mathrm{VFH}}$).

#### 4.5.3. Key Findings

1. **Comprehensive superior performance**: Table 5 shows that LDS-Hilbert outperforms all comparison methods across nearly all metrics. Compared to SRS, the LDS-Hilbert method achieves approximately 1.03 times better performance on the Point-to-Mesh and Chamfer Distance metrics, indicating better overall geometric fidelity. Against the FPS and Voxel methods, the advantage is even more pronounced, with Performance Ratios reaching 1.46 and 1.22, respectively, for the Point-to-Mesh Distance, and 1.16 and 1.07 for the Chamfer Distance.
2. **Trade-off in coverage vs. structure preservation**: While the FPS and Voxel methods show lower Hausdorff Distance from ground truth to sample ($D_{H,95,g2s}$) compared to LDS-Hilbert (Performance Ratios of 0.93 and 0.89, respectively), this advantage comes at the cost of poorer performance in other metrics. This pattern, consistent with our findings in Experiment 2, confirms that these methods prioritize uniform coverage at the expense of preserving the original geometric structure.
3. **Superior morphological fidelity**: For the VFH Distance, which captures overall shape similarities, LDS-Hilbert demonstrates substantial advantages over all comparison methods, with Performance Ratios of 1.18, 1.21, 1.44, and 1.17 against SRS, FPS, Voxel, and LDS-PCA, respectively. This indicates that LDS-Hilbert preserves the essential morphological characteristics of these complex models significantly better than other methods.
4. **Consistent Win Percentages**: Table 6 further supports LDS-Hilbert’s superior performance, showing that it outperforms SRS in approximately 61.54% of cases for the Point-to-Mesh Distance and 100% for the Chamfer Distance. Against FPS and Voxel, LDS-Hilbert wins in 100% and 79.49% of cases, respectively, for the Point-to-Mesh Distance. For the comprehensive VFH metric, LDS-Hilbert achieves impressive win rates of 89.74%, 66.67%, 66.67%, and 87.18% against SRS, FPS, Voxel, and LDS-PCA, respectively.
5. **Value of Hilbert ordering**: The comparison with LDS-PCA isolates the contribution of the Hilbert curve ordering. While LDS-PCA performs relatively well compared to other methods, LDS-Hilbert consistently outperforms it across most metrics, with particularly notable advantages in the Point-to-Mesh Distance (58.97% win rate) and VFH Distance (87.18% win rate). This confirms that the Hilbert curve’s spatial coherence properties specifically enhance the reconstruction quality for complex models.

For the corresponding significance test, please refer to Section 4.7.

#### 4.5.4. Implications and Synthesis

Given the diversity of the ModelNet40 dataset, which includes objects ranging from simple geometric forms to complex multi-component assemblies, these results demonstrate that LDS-Hilbert’s advantages extend beyond the basic parametric forms and closed prismatic geometries tested in previous experiments. The method appears particularly effective at handling the varied density distributions and complex feature arrangements found in real-world CAD models.

The primary advantage of LDS-Hilbert in this experiment appears to be its ability to maintain a balance between preserving local features (such as sharp edges and corners) while also ensuring sufficient point density in smooth regions. This balanced approach results in reconstructions that better capture both the overall structure and the fine details of the original models, making it particularly suitable for complex digital twin applications where fidelity across multiple scales is essential.

These findings align with the theoretical foundation of our method: the Hilbert curve’s locality preservation combined with the Low-Discrepancy Sequence’s uniform coverage creates a synergistic approach that offers particular advantages for complex, multi-feature objects. The strong performance across the diverse ModelNet40 dataset suggests that LDS-Hilbert provides a robust, generally applicable solution for high-fidelity down-sampling of 3D point clouds representing real-world objects.

### 4.6. Experiment 4: Mesh Reconstruction from Real-World Laser Scans (Stanford Dataset)

This experiment tested the down-sampling methods on challenging real-world data obtained from laser scans. These data featured complex organic shapes, intricate surface details, non-uniform point distributions, and potential scanning imperfections. The dataset consisted of 254 individual scans from four well-known models in the Stanford 3D Scanning Repository (Happy Buddha, Bunny, Dragon, Armadillo). The provided high-resolution meshes served as the ground truth, with their vertices forming the ground truth point clouds. Input point clouds were generated by adding Gaussian noise to these ground truth points. Each method down-sampled these clouds to appropriate ratios—20% for Armadillo and Dragon, and 10% for Happy Buddha and Bunny—to account for differences in model complexity. The resulting down-sampled point clouds were used to reconstruct Type C Mesh digital twins via the Ball Pivoting algorithm. Fidelity was assessed using the identical set of geometric metrics employed in previous experiments.

#### 4.6.1. Visual Assessment

**Figure 5:**
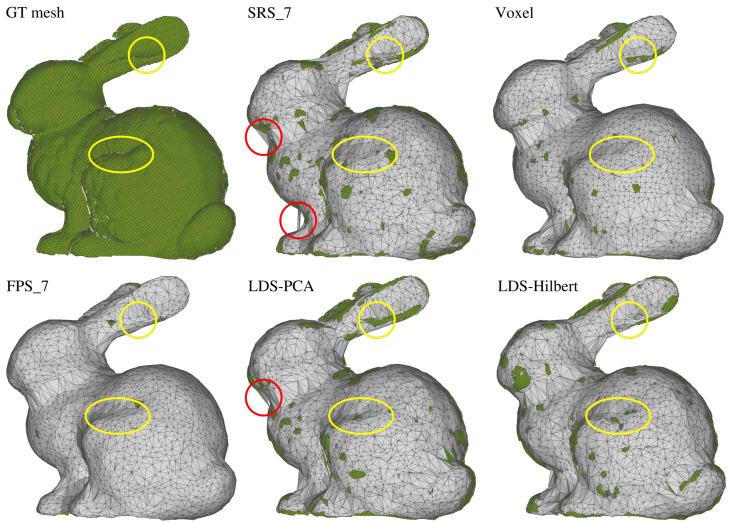
Comparative analysis of bunny model reconstruction. The yellow circles highlight two important detail regions and the red circles indicate significant surface defects.

Figure 5 provides a detailed visual comparison of reconstruction quality using the Stanford Bunny as an example. Rather than showing the reconstructions separately, this visualization overlays the ground truth mesh (in green) with the reconstructed meshes (in gray) from each method. This approach clearly reveals where reconstructions deviate from the original model.

Several important observations can be made from this visualization:

1. The SRS, LDS-PCA, and LDS-Hilbert methods produce meshes that interlock with the ground truth mesh, indicating their overall dimensions closely match the original. In contrast, the FPS and Voxel methods generate meshes that completely envelop the ground truth, suggesting systematic overestimation of the model dimensions.
2. The yellow circles highlight two important detail regions: the ear and knee indentations of the bunny model. The LDS-Hilbert method successfully preserves these concave details, while the SRS, FPS, and Voxel methods tend to “smooth over” these features, losing important geometric characteristics. This observation confirms LDS-Hilbert’s superior ability to maintain fine details in organic, complex models.
3. Red circles on the SRS and LDS-PCA reconstructions indicate significant surface defects and irregularities not present in the ground truth. These artifacts are largely absent in the LDS-Hilbert reconstruction, which achieves a better balance between detail preservation and surface smoothness.

The visual differences in detail preservation highlighted in Figure 5 are further substantiated by the quantitative metrics presented in Table 7 and Table 8, which we will now discuss.

#### 4.6.2. Quantitative Results

Table 7 and Table 8 present the Performance Ratio and Win Percentage metrics for each evaluation criterion. The Performance Ratio (Table 7) quantifies the relative error of each comparison method to LDS-Hilbert, with values greater than 1.0 indicating LDS-Hilbert’s superior performance. The Win Percentage (Table 8) shows the proportion of test cases where LDS-Hilbert outperformed each comparison method.

#### 4.6.3. Key Findings

1. **Superior shape preservation**: The most striking result appears in the VFH Distance ($D_{\mathrm{VFH}}$) metric, where LDS-Hilbert dramatically outperforms all comparison methods with Performance Ratios of 2.60, 3.19, 3.25, and 2.41 against SRS, FPS, Voxel, and LDS-PCA, respectively. These substantial differences indicate that LDS-Hilbert preserves the essential morphological characteristics and overall shape of these complex organic models 2–3 times better than other methods. The Win Percentages for this metric range from 91.67% to 97.92%, demonstrating consistent superiority across nearly all test cases.
2. **Balanced geometric fidelity**: The Point-to-Mesh Distance ($D_{p2m}$) shows LDS-Hilbert achieving Performance Ratios of 1.00, 1.47, 1.16, and 1.00 against SRS, FPS, Voxel, and LDS-PCA, respectively. While the advantages over SRS and LDS-PCA appear modest when averaged, the Win Percentages of 41.67% and 60.42% reveal that LDS-Hilbert outperforms these methods on many individual scans. Against FPS and Voxel, the advantages are more pronounced, with Win Percentages of 97.92% and 85.42%.
3. **Comprehensive distance metrics**: For the Chamfer Distance ($D_{\mathrm{CD}}$), which considers both how well sampled points represent the surface and how well the surface is covered by sampled points, LDS-Hilbert demonstrates Performance Ratios of 1.02, 1.17, 1.02, and 1.02 against SRS, FPS, Voxel, and LDS-PCA, respectively. The Win Percentages for this metric are 89.58%, 100%, 43.75%, and 85.42%, indicating particularly consistent advantages over FPS and strong performance against SRS and LDS-PCA.
4. **Trade-off in coverage metrics**: For the Hausdorff Distance from ground truth to sample ($D_{H,95,g2s}$), the FPS and Voxel methods achieve lower error rates than LDS-Hilbert, with Performance Ratios of 0.87 and 0.83, respectively. This reflects these methods’ design focus on ensuring uniform coverage at the expense of other quality aspects. The 0% Win Percentage against these methods confirms this is a systematic pattern. However, LDS-Hilbert performs significantly better on the Hausdorff Distance from sample to ground truth ($D_{H,95,s2g}$), with Performance Ratios of 1.00, 1.28, 1.13, and 1.00 against SRS, FPS, Voxel, and LDS-PCA, respectively.
5. **Value of Hilbert ordering**: The comparison with LDS-PCA isolates the contribution of the Hilbert curve ordering. While LDS-PCA performs reasonably well across most metrics, LDS-Hilbert maintains an advantage in several key areas, particularly in the VFH Distance (Performance Ratio of 2.41) and Win Percentage (91.67%). This confirms that the Hilbert curve’s spatial coherence properties specifically enhance shape preservation for complex organic models.

The significance analysis is provided in Section 4.7.

#### 4.6.4. Implications and Synthesis

These results on the Stanford dataset—widely recognized as a benchmark for 3D reconstruction algorithms—provide compelling evidence that the LDS-Hilbert method’s advantages extend to real-world scanned data. The method’s ability to maintain fine details while preserving overall shape integrity makes it particularly valuable for digital twin applications involving complex, organic geometries with varying feature scales and density distributions.

The Stanford dataset results are especially meaningful because these models represent genuine scanned data with all the complexities and imperfections inherent in real-world acquisition processes. LDS-Hilbert’s robust performance on this data suggests it would be well suited for practical applications in cultural heritage preservation, medical modeling, and other fields where high-fidelity digital reproduction of complex organic forms is essential.

The most significant advantage of LDS-Hilbert appears to be in preserving the overall morphological characteristics of the models, as evidenced by the dramatic improvements in the VFH Distance. This suggests that the method is particularly valuable for applications where the visual appearance and geometric features of the digital twin must closely match the physical original.

Combined with our findings from previous experiments, these results establish LDS-Hilbert as a consistently superior approach for point cloud down-sampling across a wide range of geometry types, from simple parametric surfaces to complex real-world scans. The method’s advantages become increasingly pronounced as object complexity increases, making it especially relevant for challenging digital twin applications where both computational efficiency and geometric fidelity are critical requirements.

### 4.7. Statistical Significance

We conducted paired *t*-tests comparing LDS-Hilbert against each alternative method across all experiments. The quantities to be tested are the distance metrics in each experiment. The computed t-values are summarized in Table 9, Table 10, Table 11 and Table 12. The corresponding significance levels are also indicated, where * denotes p<0.05 and ** denotes p<0.01.

The paired *t*-tests reveal that LDS-Hilbert’s performance advantage becomes more pronounced as the point cloud size *N* increases, particularly evident in Experiments 1 and 2. This suggests that LDS-Hilbert’s ability to maintain geometric fidelity is more effective for larger datasets. In the more complex point clouds of Experiments 3 and 4, LDS-Hilbert consistently outperforms other methods, with particularly notable improvements in Experiment 4, which features intricate surfaces. Across all mesh reconstruction tasks, LDS-Hilbert shows significant reductions in the VFH Distance, indicating superior preservation of overall shape and structural details. Additionally, LDS-Hilbert demonstrates balanced performance in other metrics, such as $D_{p2m}$ and $D_{\mathrm{CD}}$, suggesting its effectiveness in maintaining both local and global fidelity. These results highlight LDS-Hilbert’s robustness in handling diverse datasets and its potential for enhancing digital twin fidelity in various applications.

#### Summary of Computational Efficiency

**Figure A3:**
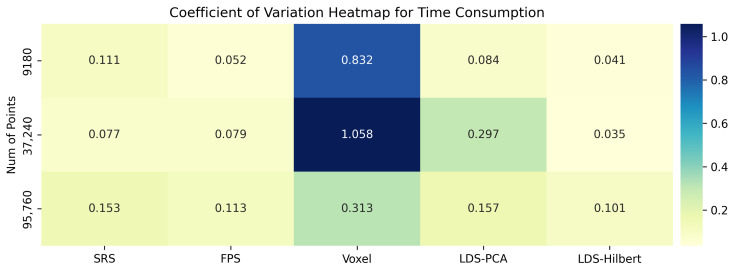
Coefficient of variation heatmap for time consumption.

Beyond fidelity, computational efficiency is a practical consideration for down-sampling methods. A comparative analysis of the average down-sampling time and its coefficient of variation was conducted (details in Appendix E, Table A4, Figure A3). The results show that the proposed LDS-Hilbert method exhibits practical execution times comparable to other established techniques like Farthest Point Sampling (FPS) and the LDS-PCA variant used in our ablation study. While not as computationally inexpensive as Simple Random Sampling (SRS) or optimized Voxel Grid implementations (which benefit from simple random selection or grid hashing, respectively), LDS-Hilbert demonstrates highly stable performance with low variance in execution time across different runs and geometries. This predictability is a notable advantage over the Voxel method, whose timing and output size can be sensitive to the chosen cell size parameter. This balance of consistently high fidelity (demonstrated across Experiments 1–4) and predictable, reasonable computational cost further supports the suitability of LDS-Hilbert for practical digital twin applications where both accuracy and performance stability are valued.

## 5. Conclusions and Limitations

### 5.1. Conclusions

This research addresses the challenge of point cloud down-sampling through the development of a novel LDS-Hilbert approach. Our method leverages the complementary mathematical properties of Low-Discrepancy Sequences and Hilbert curve ordering to create a synergistic approach that preserves both global structure and local details.

Across four diverse experiments, LDS-Hilbert consistently outperformed traditional approaches:

1. For parametric fitting (Type A), our method achieved parameter recovery with up to 40% lower error.
2. For mesh reconstruction (Types B and C), it demonstrated superior preservation of critical features while maintaining better overall shape fidelity, with VFH Distances up to 50% better.
3. On real-world laser scans, LDS-Hilbert preserved fine details that other methods smoothed over or distorted.

The integration of Low-Discrepancy Sequences with Hilbert curve ordering represents a significant advancement in point cloud down-sampling technology. The method achieves these improvements without requiring feature-specific calculations, extensive pre-processing, or task-specific training data.

Beyond computational efficiency, this work has significant implications across various domains. It can improve environmental perception for autonomous vehicles, enhance structural assessment in infrastructure digital twins, and potentially boost diagnostic accuracy in medical imaging by better preserving crucial details. Importantly, by maintaining the original density distributions, our method respects the inherent information captured during scanning, often reflecting focused attention on complex or critical regions.

Building upon these findings, we offer practical guidelines for method selection in Section 5.1 and outline several avenues for future research in Section 5.2 to further advance this work.

#### Practical Guidelines for Method Selection

Based on our experimental results, we offer the following guidelines for practitioners:

1. **For large-scale point clouds (>100 K points)**: LDS-Hilbert consistently offers superior performance across all tested geometry types and is strongly recommended, particularly when down-sampling ratios below 20% are required.
2. **For time-critical applications**: If processing speed is the primary concern, SRS provides the fastest execution but with quality compromises. Voxel Grid Filtering offers a reasonable compromise between speed and quality for simpler geometries without sharp features.
3. **For feature-rich models**: When the point cloud contains important sharp features, edges, or corners, LDS-Hilbert significantly outperforms traditional methods, with differences most pronounced for complex CAD models and real-world scans.
4. **For parametric surface fitting**: When the goal is parameter recovery rather than visual fidelity, the advantage of LDS-Hilbert over FPS and Voxel methods is particularly substantial, with error reductions exceeding 40%.
5. **Implementation considerations**: The LDS-Hilbert method requires minimal parameter tuning beyond specifying the desired output size, making it more robust across different point cloud characteristics compared to methods like FPS (neighborhood size) or Voxel (cell size) that require careful parameter selection.

### 5.2. Limitations and Future Work

Despite its advantages, several limitations warrant acknowledgment:

1. The current implementation has been validated primarily on 3D point clouds; extension to higher-dimensional feature spaces represents an important area for future investigation.
2. The approach treats all regions with equal importance; future work could explore adaptive variants incorporating importance weighting.
3. Integration with learning-based methods and adaptation for streaming or online processing could enhance applicability.
4. The current study provides average processing times, but a dedicated analysis of real-time performance (e.g., latency, throughput) on large-scale point clouds, crucial for certain applications, remains an area for future investigation.
5. Future work includes establishing formal theoretical bounds on LDS-Hilbert’s approximation error. This involves analyzing how Hilbert curve locality and LDS discrepancy jointly determine the sampled subset’s representativeness (geometric fidelity and uniformity), offering stronger guarantees for critical applications.

By addressing these limitations, the method’s utility can be further expanded to benefit a wider range of applications in fields such as autonomous navigation, industrial inspection, cultural heritage preservation, and medical modeling.

## Figures and Tables

**Table 1:** Performance Ratios for LDS-Hilbert over other methods on parametric surface fidelity ($D_{L1}$).

N	Ratio	SRS	FPS	Voxel	LDS-PCA
9180	10%	1.40	2.59	2.68	1.47
	20%	0.97	1.66	1.48	0.84
37,240	10%	0.90	2.04	1.81	0.90
	20%	1.17	2.15	1.98	0.96
95,760	10%	1.29	3.07	2.97	1.15
	20%	1.20	2.54	2.50	1.18
**Average:**		1.16	2.34	2.24	1.08

**Table 2:** Win Percentage of LDS-Hilbert over other methods on parametric surface fidelity ($D_{L1}$).

N	Ratio	SRS	FPS	Voxel	LDS-PCA
9180	10%	57.14%	100.00%	100.00%	42.86%
	20%	28.57%	71.43%	71.43%	14.29%
37,240	10%	42.86%	71.43%	85.71%	42.86%
	20%	57.14%	100.00%	85.71%	57.14%
95,760	10%	100.00%	100.00%	100.00%	71.43%
	20%	100.00%	100.00%	100.00%	100.00%
**Average:**		64.29%	90.48%	90.48%	54.76%

**Table 3:** Performance Ratio on closed prismatic geometry mesh fidelity.

N	Metrics	SRS	FPS	Voxel	LDS-PCA
9180	$D_{p2m}$	1	1.39	0.89	1
	$D_{\mathrm{CD}}$	1.08	1.06	0.94	1.07
	$D_{H,95,\;s2g}$	0.99	1.24	1.04	0.99
	$D_{H,95,\;g2s}$	1.21	0.88	0.87	1.19
	$D_{\mathrm{VFH}}$	1.06	0.99	1.09	1.08
37,240	$D_{p2m}$	1	1.67	1.17	1
	$D_{\mathrm{CD}}$	1	1.2	0.99	0.99
	$D_{H,95,\;s2g}$	0.99	1.36	1.16	0.99
	$D_{H,95,\;g2s}$	1	0.84	0.78	0.99
	$D_{\mathrm{VFH}}$	1.13	1.05	1.04	1.11
95,760	$D_{p2m}$	1	1.64	1.36	1
	$D_{\mathrm{CD}}$	1.04	1.33	1.18	1.04
	$D_{H,95,\;s2g}$	1	1.39	1.26	1.01
	$D_{H,95,\;g2s}$	1.14	1.04	0.97	1.14
	$D_{\mathrm{VFH}}$	1.96	1.96	1.85	1.86
**Average:**	$D_{p2m}$	1	1.57	1.14	1
	$D_{\mathrm{CD}}$	1.04	1.2	1.04	1.04
	$D_{H,95,\;s2g}$	1	1.33	1.15	1
	$D_{H,95,\;g2s}$	1.12	0.92	0.88	1.11
	$D_{\mathrm{VFH}}$	1.39	1.33	1.33	1.35

**Table 4:** Win Percentage on closed prismatic geometry mesh fidelity.

N	Metrics	SRS	FPS	Voxel	LDS-PCA
9180	$D_{p2m}$	46.67%	100.00%	6.67%	40.00%
	$D_{\mathrm{CD}}$	100.00%	93.33%	0.00%	100.00%
	$D_{H,95,\;s2g}$	26.67%	100.00%	53.33%	46.67%
	$D_{H,95,\;g2s}$	100.00%	0.00%	0.00%	100.00%
	$D_{\mathrm{VFH}}$	60.00%	46.67%	86.67%	73.33%
37,240	$D_{p2m}$	40.00%	100.00%	93.33%	46.67%
	$D_{\mathrm{CD}}$	20.00%	100.00%	40.00%	13.33%
	$D_{H,95,\;s2g}$	33.33%	100.00%	100.00%	26.67%
	$D_{H,95,\;g2s}$	26.67%	0.00%	0.00%	26.67%
	$D_{\mathrm{VFH}}$	66.67%	73.33%	33.33%	46.67%
95,760	$D_{p2m}$	66.67%	100.00%	100.00%	40.00%
	$D_{\mathrm{CD}}$	100.00%	100.00%	100.00%	100.00%
	$D_{H,95,\;s2g}$	40.00%	100.00%	100.00%	60.00%
	$D_{H,95,\;g2s}$	100.00%	100.00%	13.33%	100.00%
	$D_{\mathrm{VFH}}$	100.00%	100.00%	86.67%	100.00%
**Average:**	$D_{p2m}$	51.00%	100.00%	67.00%	42.00%
	$D_{\mathrm{CD}}$	73.00%	98.00%	47.00%	71.00%
	$D_{H,95,\;s2g}$	33.00%	100.00%	84.00%	44.00%
	$D_{H,95,\;g2s}$	76.00%	33.00%	4.00%	76.00%
	$D_{\mathrm{VFH}}$	76.00%	73.00%	69.00%	73.00%

**Table 5:** Performance Ratio on ModelNet40 mesh fidelity.

Metrics	SRS	FPS	Voxel	LDS-PCA
$D_{p2m}$	1	1.46	1.22	1
$D_{\mathrm{CD}}$	1.03	1.16	1.07	1.03
$D_{H,95,\;s2g}$	1	1.27	1.19	1
$D_{H,95,\;g2s}$	1.08	0.93	0.89	1.08
$D_{\mathrm{VFH}}$	1.18	1.21	1.44	1.17

**Table 6:** Win Percentage on ModelNet40 mesh fidelity.

Metrics	SRS	FPS	Voxel	LDS-PCA
$D_{p2m}$	61.54%	100.00%	79.49%	58.97%
$D_{\mathrm{CD}}$	100.00%	97.44%	71.79%	97.44%
$D_{H,95,\;s2g}$	48.72%	100.00%	97.44%	53.85%
$D_{H,95,\;g2s}$	100.00%	2.56%	0.00%	100.00%
$D_{\mathrm{VFH}}$	89.74%	66.67%	66.67%	87.18%

**Table 7:** Performance Ratio on Stanford scans fidelity.

Metrics	SRS	FPS	Voxel	LDS-PCA
$D_{p2m}$	1	1.47	1.16	1
$D_{\mathrm{CD}}$	1.02	1.17	1.02	1.02
$D_{H,95,\;s2g}$	1	1.28	1.13	1
$D_{H,95,\;g2s}$	1.08	0.87	0.83	1.07
$D_{\mathrm{VFH}}$	2.6	3.19	3.25	2.41

**Table 8:** Win Percentage on Stanford scans fidelity.

Metrics	SRS	FPS	Voxel	LDS-PCA
$D_{p2m}$	41.67%	97.92%	85.42%	60.42%
$D_{\mathrm{CD}}$	89.58%	100.00%	43.75%	85.42%
$D_{H,95,\;s2g}$	39.58%	100.00%	100.00%	45.83%
$D_{H,95,\;g2s}$	100.00%	0.00%	0.00%	97.92%
$D_{\mathrm{VFH}}$	95.83%	97.92%	97.92%	91.67%

**Table 9:** Paired *t*-test on $D_{L1}$ in Experiment 1.

N	Ratio	SRS	FPS	Voxel	LDS-PCA
9180	0.1	2.2626	2.3988	2.3613	1.4792
	0.2	1.8992	2.0358	2.3981	2.1543
37,240	0.1	1.4637	2.2552	2.2581	0.1516
	0.2	3.4722 *	2.4084	2.1334	1.4663
95,760	0.1	4.7777 **	2.5374 *	2.6974 *	3.3146 *
	0.2	3.9261 **	2.6270 *	2.4392	4.0564 **

*: p<0.05 and **: p<0.01.

**Table 10:** Paired *t*-test on Experiment 2.

N	Metric	SRS	FPS	Voxel	LDS-PCA
9180	$D_{H,95,\;s2g}$	−0.7048	15.2714 **	1.4661	−1.5009
	$D_{H,95,\;g2s}$	17.4325 **	−13.5211	−9.4246	14.6754 **
	$D_{\mathrm{CD}}$	21.6308 **	5.5173 **	−6.7199	13.4815 **
	$D_{p2m}$	0.3618	15.6020 **	−4.8813	−0.0572
	$D_{\mathrm{VFH}}$	1.0269	−0.5189	2.0438	1.0983
37,240	$D_{H,95,\;s2g}$	−1.9041	59.0391 **	13.6449 **	−1.3476
	$D_{H,95,\;g2s}$	−2.071	−11.0762	−13.8813	−2.7027
	$D_{\mathrm{CD}}$	−2.8076	25.7126 **	−0.9972	−2.7011
	$D_{p2m}$	−1.2783	82.7670 **	8.7161 **	0.0613
	$D_{\mathrm{VFH}}$	1.6435	0.9146	−0.0662	1.5344
95,760	$D_{H,95,\;s2g}$	0.6534	116.4929 **	28.0707 **	2.4064 *
	$D_{H,95,\;g2s}$	20.8601 **	20.9876 **	−3.5097	19.7301 **
	$D_{\mathrm{CD}}$	21.0916 **	91.6320 **	18.7912 **	16.9903 **
	$D_{p2m}$	0.9368	140.4269 **	23.6502 **	−0.1805
	$D_{\mathrm{VFH}}$	2.9560 *	3.1374 **	2.7974 *	3.1259 **

*: p<0.05 and **: p<0.01.

**Table 11:** Paired *t*-test on Experiment 3.

Metric	SRS	FPS	Voxel	LDS-PCA
$D_{H,95,\;s2g}$	−0.4106	25.3279 **	1.3399	−0.219
$D_{H,95,\;g2s}$	3.9070 **	−1.3709	−1.3842	4.7496 **
$D_{\mathrm{CD}}$	4.0613 **	0.7314	1.2208	4.5332 **
$D_{p2m}$	0.9432	25.1428 **	7.0922 **	0.7867
$D_{\mathrm{VFH}}$	3.6487 **	2.1050 *	2.4367 *	3.2510 **

*: p<0.05 and **: p<0.01.

**Table 12:** Paired *t*-test on Experiment 4.

Metric	SRS	FPS	Voxel	LDS-PCA
$D_{H,95,\;s2g}$	−1.2756	53.5738 **	15.8549 **	−0.5698
$D_{H,95,\;g2s}$	8.2243 **	−9.69	−11.5884	8.3965 **
$D_{\mathrm{CD}}$	7.6155 **	28.5556 **	−0.0294	7.5771 **
$D_{p2m}$	−1.9121	3.9056 **	2.3743 *	0.5398
$D_{\mathrm{VFH}}$	13.2551 **	18.6591 **	18.6035 **	9.1917 **

*: p<0.05 and **: p<0.01.

**Figure A1:**
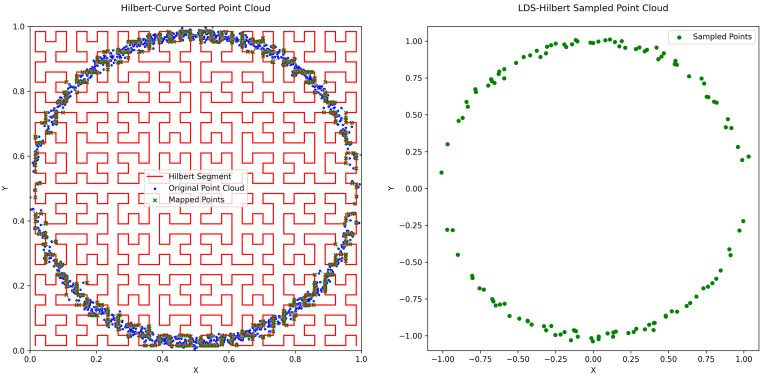
Visualization of the down-sampled point cloud using LDS-Hilbert.

**Figure A2:**
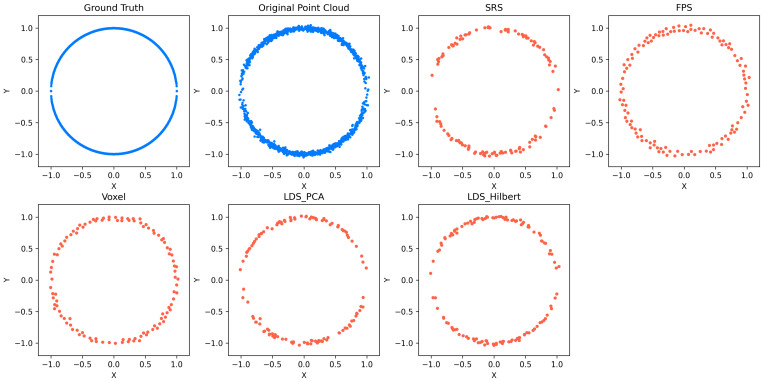
Comparison of down-sampling results using different methods. The point cloud downsampled by the LDS-Hilbert method better preserves the distributions of both the ground truth and the original point cloud.

## Data Availability

The key scripts and data supporting the experiments in this paper are openly available in the LDS-Hilbert-Point-Cloud repository at https://github.com/Yuening-Ma/LDS-Hilbert-Point-Cloud (accessed on 9 June 2025).

## Appendix A. Illustration of Proposed Method

To demonstrate our method’s effectiveness, we present a 2D point cloud example consisting of 1230 points arranged in a circular contour with Gaussian noise (σ=0.02) added. Due to the sampling methodology, the point distribution is denser near the Y-axis and sparser away from it.

Figure A1 illustrates the sampling process in LDS-Hilbert. Figure A2 compares our method with benchmark methods (SRS, FPS, Voxel) at a 10% sampling ratio.

Both FPS and Voxel produce spatially uniform results but alter the original density variations. The LDS-Hilbert method preserves the original spatial distribution, including density differences. While SRS generally adheres to the true distribution, its stochastic nature can lead to local gaps or clusters.

For a more precise quantitative comparison of how well each down-sampling method preserves the point cloud’s morphology, we calculated the following metrics comparing the down-sampled cloud $P^{\prime }$ to the ground truth cloud $P_{\mathrm{true}}$: $D_{\mathrm{pc}}\left(P^{\prime },P_{\mathrm{true}}\right)$ (average distance from sampled points to the nearest true point), $D_{\mathrm{pc}}\left(P_{\mathrm{true}},P^{\prime }\right)$ (average distance from true points to the nearest sampled point), and the Chamfer Distance $D_{\mathrm{Chamfer}}\left(P_{\mathrm{true}},P^{\prime }\right)$ (the sum of the previous two metrics). As Viewpoint Feature Histograms (VFH) are not applicable to 2D data, we used the Kullback–Leibler (KL) divergence between distribution histograms of $P^{\prime }$ and $P_{\mathrm{true}}$ to compare the preservation of the overall point cloud structure. Table A1 presents these results, in which a ratio >1 means LDS-Hilbert achieves better performance.

**Table A1:** Performance Ratio of down-sampling methods on the 2D example using fidelity metrics.

Metrics	SRS	FPS	Voxel	LDS-PCA
$D_{\mathrm{pc}}\left(P^{\prime },P_{\mathrm{true}}\right)$	1.2	1.99	1.26	0.98
$D_{\mathrm{pc}}\left(P_{\mathrm{true}},P^{\prime }\right)$	1.23	1.23	1	1.12
$D_{\mathrm{CD}}$	1.22	1.52	1.1	1.07
$D_{\mathrm{KL}}\left(\cdot ∥\cdot \right)$	1.86	2.39	2.64	2.11

## Appendix B. Dataset Generation and Parameter Fitting in Experiment 1

Point clouds in Experiment 1 were generated from seven analytical surfaces with specific parameters in Table A2.

Gaussian noise (σ=0.05 relative to object scale) was added to simulate measurement errors.

The parametric surface fitting is with least-squares optimization. The optimization objectives are summarized in Table A2 as well.

Plane fitting is implemented using the least squares API in NumPy, while sphere, ellipsoid, torus, cylinder, cone, and paraboloid fitting are implemented using the corresponding API in the SciPy library.

**Table A2:** Surface types, parameters, and optimization objectives.

No.	Surface Type	Model Equation	Parameters	Optimization Objective
1	Plane	$\left\{\begin{array}{c} z=ax+by+c\\ x\in [-2,2],y\in [-2,2] \end{array}\right.$	$\left\{\begin{array}{c} a=1\\ b=0.5\\ c=1.5 \end{array}\right.$	z−(ax+by+c) = 0
2	Sphere	$\left\{\begin{array}{c} x=x_{c}+r\sin\phi \cos w\\ y=y_{c}+r\sin\phi \sin w\\ z=z_{c}+r\cos\phi \\ w\in [0,2\pi ],\phi \in [0,\pi ] \end{array}\right.$	$\left\{\begin{array}{c} x_{c}=0\\ y_{c}=0\\ z_{c}=0\\ r=1 \end{array}\right.$	$\sqrt{{\left(x-x_{c}\right)}^{2}+{\left(y-y_{c}\right)}^{2}+{\left(z-z_{c}\right)}^{2}}-r$ = 0
3	Ellipsoid	$\left\{\begin{array}{c} x=x_{c}+a\sin\phi \cos w\\ y=y_{c}+b\sin\phi \sin w\\ z=z_{c}+c\cos\phi \\ w\in [0,2\pi ],\phi \in [0,\pi ] \end{array}\right.$	$\left\{\begin{array}{c} x_{c}=0\\ y_{c}=0\\ z_{c}=0\\ a=3\\ b=2\\ c=1 \end{array}\right.$	$\frac{{\left(x-x_{c}\right)}^{2}}{a^{2}}+\frac{{\left(y-y_{c}\right)}^{2}}{b^{2}}+\frac{{\left(z-z_{c}\right)}^{2}}{c^{2}}-1$ = 0
4	Torus	$\left\{\begin{array}{c} x=x_{c}+\left(R+r\cos\phi \right)\cos w\\ y=y_{c}+\left(R+r\cos\phi \right)\sin w\\ z=z_{c}+r\sin\phi \\ w\in [0,2\pi ],\phi \in [0,2\pi ] \end{array}\right.$	$\left\{\begin{array}{c} x_{c}=0\\ y_{c}=0\\ z_{c}=0\\ R=2\\ r=0.5 \end{array}\right.$	$\sqrt{{\left(\sqrt{{\left(x-x_{c}\right)}^{2}+{\left(y-y_{c}\right)}^{2}}-R\right)}^{2}+{\left(z-z_{c}\right)}^{2}}$ − *r* = 0
5	Cylinder	$\left\{\begin{array}{c} x=x_{c}+r\cos w\\ y=y_{c}+r\sin w\\ z\in [-2,2]\\ w\in [0,2\pi ] \end{array}\right.$	$\left\{\begin{array}{c} x_{c}=0\\ y_{c}=0\\ r=1 \end{array}\right.$	$\sqrt{{\left(x-x_{c}\right)}^{2}+{\left(y-y_{c}\right)}^{2}}-r$ = 0
6	Cone	$\left\{\begin{array}{c} x=x_{c}+rz\cos w\\ y=y_{c}+rz\sin w\\ z\in [0,2]\\ w\in [0,2\pi ] \end{array}\right.$	$\left\{\begin{array}{c} x_{c}=0\\ y_{c}=0\\ z_{c}=0\\ r=1 \end{array}\right.$	$\sqrt{{\left(x-x_{c}\right)}^{2}+{\left(y-y_{c}\right)}^{2}}-r$ = 0
7	Paraboloid	$\left\{\begin{array}{c} x=x_{c}+r\cos w\\ y=y_{c}+r\sin w\\ z=z_{c}+ar^{2}\\ r\in [0,2],w\in [0,2\pi ] \end{array}\right.$	$\left\{\begin{array}{c} x_{c}=0\\ y_{c}=0\\ z_{c}=0\\ a=0.5 \end{array}\right.$	$z-[a{\left(x-x_{c}\right)}^{2}+a{\left(y-y_{c}\right)}^{2}+z_{c}]$ = 0

## Appendix C. Dataset Generation for Closed Geometries

Experiment 2 used 15 geometric shapes with dimensions defined relative to a base scale factor (SCALE = 100), whose dimensions are shown in Table A3.

Each shape was randomly rotated before sampling points using Poisson disk sampling. Gaussian noise (σ=1) was added to simulate scanning errors.

**Table A3:** Geometric shapes and their parameters. Note that the numerical values need to be multiplied by a base scale factor (SCALE = 100).

ID	Shape	Param_1	Param_2	Param_3
1	Sphere	Radius r=0.5	-	-
2	Cylinder	Radius r=0.3	Height h=1	-
3	Equilateral Triangular Prism	Base edge length a=0.6	Height h=1	-
4	Cube	Edge length a=1	-	-
5	Rectangular Prism	Length l=0.4	Width w=0.8	Height h=1
6	Pentagonal Prism	Base edge length a=0.4	Height h=1	-
7	Hexagonal Prism	Base edge length a=0.4	Height h=1	-
8	L-beam	Width w=0.6	Height h=1	Thickness t=0.1
9	T-beam	Width w=0.6	Height h=1	Thickness t=0.1
10	U-beam	Width w=0.6	Height h=1	Thickness t=0.1
11	H-beam	Width w=0.6	Height h=1	Thickness t=0.1
12	Cone	Radius r=0.5	Height h=1	-
13	Torus	Major radius R=0.35	Minor radius r=0.15	-
14	Tetrahedron	Circumscribed radius r=0.75	-	-
15	Octahedron	Circumscribed radius r=0.5	-	-

## Appendix D. Parameter Settings for Ball Pivoting

In Experiments 2, 3, and 4, we utilized the Ball Pivoting algorithm for mesh reconstruction from point clouds. This algorithm constructs meshes by rolling a ball over the point cloud, with the radius of the ball influencing the scale of detected geometric features. To ensure consistent and effective mesh reconstruction across different point cloud densities, we dynamically adjusted the ball radius based on the average point spacing of the input data.

Specifically, we first calculated a base radius *d* that is inversely proportional to the average point spacing. Given that the point clouds represent hollow surface models with uniform heights and similar total surface areas, the average point spacing is inversely related to the square root of the number of points N. Based on this relationship, we estimated the base radius d using the following formula:

$$d=\mathrm{round}\left(\sqrt{\frac{27,000}{N}},1\right)$$

where *N* is the number of points in the point cloud. The constant 27,000 was determined through preliminary experiments to ensure that point clouds down-sampled by different methods could all generate a sufficient number of triangles, thereby guaranteeing the fairness and effectiveness of the experiments.

For Experiment 2, the radii list used was 2d, 3d, 4d, 5d, 6d. For Experiments 3 and 4, the radii list was adjusted 2d, 2.25d, 2.5d, 2.75d, 3d.

These radii lists were passed to the ball-pivoting API in the Open3d library, which attempts to create triangles using each radius in ascending order. This approach allows the algorithm to capture geometric features at different scales, ensuring accurate and complete mesh reconstruction.

By employing this dynamic radius adjustment strategy, we effectively balanced the detail retention and computational efficiency of mesh reconstruction, providing high-quality mesh models for subsequent digital twin creation.

## Appendix E. Time Consuming Analysis

To validate the efficiency of the LDS-Hilbert method, a down-sampling time analysis was conducted on the point clouds generated in Experiment 2. Specifically, the average down-sampling time and the coefficient of variation (i.e., the ratio of the standard deviation to the mean) for each method were calculated across 15 experimental objects with the same number of points. Table A4 presents the average time results, and Figure A3 shows the heatmap of the coefficients of variation.

From Table A4 and Figure A3, we can observe the following:

1. In terms of time consumption, SRS and Voxel are the most efficient methods, while FPS, LDS-PCA, and LDS-Hilbert have similar time costs. Among them, LDS-Hilbert is slightly slower, but the difference is not significant.
2. Except for the Voxel method, all down-sampling methods, including the proposed LDS-Hilbert, have low coefficients of variation in time, indicating stable efficiency. One reason for the instability of the Voxel method is that the number of output points from down-sampling is difficult to determine, often requiring adjustments to the Voxel size to achieve an output point count close to the desired number. This is also one of the drawbacks of the Voxel method.

Since the Voxel, FPS, and LDS-Hilbert algorithms all rely on well-established algorithm libraries in the industry, their time costs are influenced by the level of algorithm optimization. Therefore, this experiment is for reference only, to demonstrate that the proposed LDS-Hilbert method can achieve efficiency and stability comparable to commonly used industry algorithms.

**Table A4:** Average down-sampling time on Experiment 2 data.

N	SRS	FPS	Voxel	LDS-PCA	LDS-Hilbert
9180	0.000302	0.017179	0.002178	0.008097	0.022676
37,240	0.001111	0.056052	0.003589	0.041993	0.067687
95,760	0.003005	0.159396	0.006999	0.120169	0.197373

## References

[1] Grieves M. (2014). Digital twin: Manufacturing excellence through virtual factory replication. White Pap..

[2] Tao F., Cheng J., Qi Q., Zhang M., Zhang H., Sui F. (2018). Digital twin-driven product design, manufacturing and service with big data. Int. J. Adv. Manuf. Technol..

[3] Li D., Wei Y., Zhu R. (2023). A comparative study on point cloud down-sampling strategies for deep learning-based crop organ segmentation. Plant Methods.

[4] Lyu W., Ke W., Sheng H., Ma X., Zhang H. (2024). Dynamic Downsampling Algorithm for 3D Point Cloud Map Based on Voxel Filtering. Appl. Sci..

[5] Liu J., Li J., Wang K., Guo H., Yang J., Peng J., Xu K., Liu X., Guo J. LTA-PCS: Learnable Task-Agnostic Point Cloud Sampling. Proceedings of the 2024 IEEE/CVF Conference on Computer Vision and Pattern Recognition (CVPR).

[6] Mchirgui N., Quadar N., Kraiem H., Lakhssassi A. (2024). The Applications and Challenges of Digital Twin Technology in Smart Grids: A Comprehensive Review. Appl. Sci..

[7] Xu M. (2021). BDSR: A Best Discrepancy Sequence-Based Point Cloud Resampling Framework for Geometric Digital Twinning. Master’s Thesis.

[8] Chen W., Zhu X., Chen G., Yu B., Avidan S., Brostow G., Cissé M., Farinella G.M., Hassner T. (2022). Efficient Point Cloud Analysis Using Hilbert Curve. Proceedings of the Computer Vision—ECCV 2022.

[9] Chen X., Wu Y., Xu W., Li J., Dong H., Chen Y. (2021). PointSCNet: Point Cloud Structure and Correlation Learning Based on Space-Filling Curve-Guided Sampling. Symmetry.

[10] Hu A., Xu K., Yin X., Wang D. (2024). LiDAR Point Cloud Simplification Strategy Utilizing Probabilistic Membership. Front. Phys..

[11] Chen L., Feng C., Ma Y., Wang C. (2024). A review of rigid point cloud registration based on deep learning. Front. Neurorobot..

[12] Liu X., Zhang Z. (2011). Effects of LiDAR Data Reduction and Breaklines on the Accuracy of Digital Elevation Model. Surv. Rev..

[13] Al-Rawabdeh A., He F., Habib A. (2020). Automated Feature-Based Down-Sampling Approaches for Fine Registration of Irregular Point Clouds. Remote Sens..

[14] Rusu R.B., Cousins S. 3D is here: Point Cloud Library (PCL). Proceedings of the IEEE International Conference on Robotics and Automation (ICRA).

[15] Schnabel R., Klein R., Botsch M., Chen B., Pauly M., Zwicker M. (2006). Octree-based Point-Cloud Compression. Proceedings of the Symposium on Point-Based Graphics.

[16] Meagher D. (1982). Geometric modeling using octree encoding. Comput. Graph. Image Process..

[17] Hornung A., Wurm K.M., Bennewitz M., Stachniss C., Burgard W. (2013). OctoMap: An efficient probabilistic 3D mapping framework based on octrees. Auton. Robot..

[18] Ester M., Kriegel H.P., Sander J., Xu X. A density-based algorithm for discovering clusters in large spatial databases with noise. Proceedings of the Second International Conference on Knowledge Discovery and Data Mining (KDD-96).

[19] Pauly M., Gross M., Kobbelt L.P. Efficient Simplification of Point-Sampled Surfaces. Proceedings of the Conference on Visualization ’02.

[20] Qi L., Xu S., Xiao J., Wang Y. (2023). Sharp feature-preserving 3d mesh reconstruction from point clouds based on primitive detection. Remote Sens..

[21] Chen S., Wang J., Pan W., Gao S., Wang M., Lu X. (2023). Towards Uniform Point Distribution in Feature-preserving Point Cloud Filtering. Comput. Vis. Media.

[22] Pathak S., Baldwin-McDonald T., Sels S., Penne R., Antonacopoulos A., Chaudhuri S., Chellappa R., Liu C.L., Bhattacharya S., Pal U. (2025). GP-PCS: One-shot Feature-Preserving Point Cloud Simplification with Gaussian Processes on Riemannian Manifolds. Proceedings of the Pattern Recognition.

[23] Matsuzaki K., Nonaka K. Point Cloud Sampling Preserving Local Geometry for Surface Reconstruction. Proceedings of the 34th British Machine Vision Conference 2023, BMVC 2023.

[24] Zhang G., Zhao W., Liu J., Liu X. (2024). REPS: Reconstruction-based Point Cloud Sampling. arXiv.

[25] Zhang R., Wu Y., Jin W., Meng X. (2023). Deep-Learning-Based Point Cloud Semantic Segmentation: A Survey. Electronics.

[26] Sugimoto R., Kanai K., Maki S., Katto J. A Point Cloud Downsampling Method Balancing Global and Local Shapes for 3D Object Classification. Proceedings of the 2025 IEEE International Conference on Consumer Electronics.

[27] Deng C., Peng Z., Chen Z., Chen R. (2023). Point cloud deep learning network based on balanced sampling and hybrid pooling. Sensors.

[28] Niederreiter H. (1992). Random Number Generation and Quasi-Monte Carlo Methods.

[29] Glasserman P. (2003). Monte Carlo Methods in Financial Engineering.

[30] Jäckel P. (2002). Monte Carlo Methods in Finance.

[31] Kuipers L., Niederreiter H. (1974). Uniform Distribution of Sequences.

[32] Halton J.H. (1960). On the efficiency of certain quasi-random sequences of points in evaluating multi-dimensional integrals. Numer. Math..

[33] Sobol’ I. (1967). On the distribution of points in a cube and the approximate evaluation of integrals. USSR Comput. Math. Math. Phys..

[34] Niederreiter H. (1988). Low-discrepancy and low-dispersion sequences. J. Number Theory.

[35] Sloan I.H., Woźniakowski H. (1998). When Are Quasi-Monte Carlo Algorithms Efficient for High Dimensional Integrals?. J. Complex..

[36] Shirley P., Edwards D., Boulos S. (2008). Monte Carlo and Quasi-Monte Carlo Methods for Computer Graphics. Monte Carlo and Quasi-Monte Carlo Methods 2006.

[37] Pharr M., Jakob W., Humphreys G. (2016). Physically Based Rendering: From Theory to Implementation.

[38] Yan D., Guo J., Wang B., Zhang X., Wonka P. (2015). A Survey of Blue-Noise Sampling and Its Applications. J. Comput. Sci. Technol..

[39] Fang K.T. Some Applications of Quasi-Monte Carlo Methods in Statistics. Proceedings of the Monte Carlo and Quasi-Monte Carlo Methods 2000.

[40] Morokoff W.J., Caflisch R.E. (1995). Quasi-Monte Carlo integration. J. Comput. Phys..

[41] Dick J., Pillichshammer F. (2010). Digital Nets and Sequences: Discrepancy Theory and Quasi-Monte Carlo Integration.

[42] Zhang M., Li M., Guo L., Liu J. (2023). A low-cost AI-empowered stethoscope and a lightweight model for detecting cardiac and respiratory diseases from lung and heart auscultation sounds. Sensors.

[43] Samet H. (1990). The Design and Analysis of Spatial Data Structures.

[44] Bader M. (2012). Space-Filling Curves: An Introduction with Applications in Scientific Computing.

[45] Hilbert D. (1891). Ueber die stetige Abbildung einer Linie auf ein Flächenstück. Math. Ann..

[46] Moon B., Jagadish H., Faloutsos C., Saltz J.H. (2001). Analysis of the clustering properties of the Hilbert space-filling curve. IEEE Trans. Knowl. Data Eng..

[47] Kamel I., Faloutsos C. Hilbert R-tree: An Improved R-tree using Fractals. Proceedings of the 20th International Conference on Very Large Data Bases.

[48] Pandhare A.A. (2023). Point2Point: A Framework for Efficient Deep Learning on Hilbert sorted Point Clouds with applications in Spatio-Temporal Occupancy Prediction. arXiv.

[49] Chen J., Yu L., Wang W. (2022). Hilbert Space Filling Curve Based Scan-Order for Point Cloud Attribute Compression. IEEE Trans. Image Process..

[50] Gotsman C., Lindenbaum M. (1996). On the metric properties of discrete space-filling curves. IEEE Trans. Image Process..

[51] Skilling J. (2004). Programming the Hilbert curve. Aip Conf. Proc..

[52] Hua L.K., Wang Y. (1981). Applications of Number Theory to Numerical Analysis.

[53] Guo L., Liu J., Lu R. (2021). Subsampling bias and the best-discrepancy systematic cross validation. Sci. China Math..

[54] Cignoni P., Scopigno R. (1998). METRO: Measuring error on simplified surfaces. Comput. Graph. Forum.

[55] Barrow H.G., Tenenbaum J.M., Bolles R.C., Wolf H.C. Parametric Correspondence and Chamfer Matching: Two New Techniques for Image Matching. Proceedings of the International Joint Conference on Artificial Intelligence.

[56] Fan H., Su H., Guibas L. A Point Set Generation Network for 3D Object Reconstruction from a Single Image. Proceedings of the 2017 IEEE Conference on Computer Vision and Pattern Recognition (CVPR).

[57] Huttenlocher D.P., Klanderman G.A., Rucklidge W.J. (1993). Comparing Images Using the Hausdorff Distance. IEEE Trans. Pattern Anal. Mach. Intell..

[58] Taha A.A., Hanbury A. (2015). Metrics for evaluating 3D medical image segmentation: Analysis, selection, and tool. BMC Med. Imaging.

[59] Rusu R.B., Blodow N., Beetz M. Fast Point Feature Histograms (FPFH) for 3D registration. Proceedings of the IEEE International Conference on Robotics and Automation (ICRA).

[60] Rusu R.B., Bradski G.R., Thibaux R., Hsu J. Fast 3D Recognition and Pose Using the Viewpoint Feature Histogram. Proceedings of the IEEE/RSJ International Conference on Intelligent Robots and Systems (IROS).

[61] Zhou Q.Y., Park J., Koltun V. (2018). Open3D: A Modern Library for 3D Data Processing. arXiv.

[62] Harris C.R., Millman K.J., van der Walt S.J., Gommers R., Virtanen P., Cournapeau D., Wieser E., Taylor J., Berg S., Smith N.J. (2020). Array programming with NumPy. Nature.

[63] Dalinky L. (2023). fpsample: Python Efficient Farthest Point Sampling (FPS) Library. https://github.com/leonardodalinky/fpsample.

[64] Princeton Laboratory for Intelligent Probabilistic Systems (2023). Numpy-Hilbert-Curve: Numpy Implementation of Hilbert Curves in Arbitrary Dimensions. https://github.com/PrincetonLIPS/numpy-hilbert-curve.

[65] Pedregosa F., Varoquaux G., Gramfort A., Michel V., Thirion B., Grisel O., Blondel M., Prettenhofer P., Weiss R., Dubourg V. (2011). Scikit-learn: Machine Learning in Python. J. Mach. Learn. Res..

[66] Virtanen P., Gommers R., Oliphant T.E., Haberland M., Reddy T., Cournapeau D., Burovski E., Peterson P., Weckesser W., Bright J. (2020). SciPy 1.0: Fundamental Algorithms for Scientific Computing in Python. Nat. Methods.

